# Randomized controlled trial of early, small-volume formula supplementation among newborns: A study protocol

**DOI:** 10.1371/journal.pone.0263129

**Published:** 2022-02-04

**Authors:** Amy Sarah Ginsburg, Augusto Braima de Sa, Victoria Nankabirwa, Raimundo Co, Joanitta Murungi, Mi-Ok Kim, Rachel Brim, Flavia Namiiro, Olive Namugga, Dennis J. Hartigan-O’Connor, Susan B. Roberts, Valerie Flaherman

**Affiliations:** 1 University of Washington, Seattle, WA, United States of America; 2 International Partnership for Human Development, Bissau, Guinea-Bissau; 3 Department of Epidemiology and Biostatistics, School of Public Health, College of Health Sciences, Makerere University, Kampala, Uganda; 4 Department of Global Public Health and Primary Care, Centre for Intervention Science in Maternal and Child Health (CISMAC), Centre for International Health, University of Bergen, Bergen, Norway; 5 Department of Epidemiology and Biostatistics, University of California, San Francisco, CA, United States of America; 6 Department of Pediatrics, University of California, San Francisco, CA, United States of America; 7 Mulago Specialized Women’s and Neonatal Hospital, Kampala, Uganda; 8 Department of Medical Microbiology and Immunology and the California National Primate Research Center, University of California, Davis, CA, United States of America; 9 Division of Experimental Medicine, University of California, San Francisco, CA, United States of America; 10 Friedman School of Nutrition Science and Policy, Tufts University, Boston, MA, United States of America; Medical Research Council, SOUTH AFRICA

## Abstract

Childhood undernutrition is a major health burden worldwide that increases childhood morbidity and mortality and causes impairment in infant growth and developmental delays that can persist into adulthood. The first weeks and months after birth are critical to the establishment of healthy growth and development during childhood. The World Health Organization recommends immediate and exclusive breastfeeding (EBF). In infants for whom EBF may not meet nutritional and caloric demands, early, daily, small-volume formula supplementation along with breastfeeding may more effectively avoid underweight wasting and stunting in early infancy than breastfeeding alone. The primary objective of this randomized controlled trial is to evaluate the efficacy of formula for 30 days among low birth weight (LBW) infants <6 hours of age and those not LBW with weights <2600 grams at 4 days of age. We will compare breastfeeding and formula (up to 59 milliliters administered daily) through 30 days of infant age vs recommendations for frequent EBF without supplementation, and test the hypothesis that formula increases weight-for-age z-score at 30 days of infant age. The trial will enroll and randomize 324 mother-infant pairs in Guinea-Bissau and Uganda, and follow them for 6 months for outcomes including growth, intestinal microbiota, breastfeeding duration, infant dietary intake, and adverse events. Conservatively estimating 20% loss to follow up, this sample size provides ≥80% power per weight stratum for intervention group comparison to detect a difference of 0.20 with respect to the outcome of WAZ at day 30. This trial was approved by the University of California, San Francisco Institutional Review Board (19–29405); the Guinea-Bissau National Committee on Ethics in Health (Comite Nacional de Etica na Saude, 075/CNES/INASA/2020); the Higher Degrees, Research and Ethics Committee of Makerere University (871); and the Uganda National Council of Science and Technology (HS1226ES). We plan to disseminate study results in peer-reviewed journals and international conferences.

**Trial registration number:** NCT04704076.

## Introduction

Infants with growth impairment have greatly increased risk of mortality from sepsis, pneumonia, diarrhea, and other infections as well as delayed development [1, 2]. Long-term effects of infant malnutrition include small stature and low body mass, immune dysfunction and increased risk of infections continuing into adulthood, and cognitive impairment, poor school performance, and reduced adult productivity [3, 4]. The first weeks after birth are critical to the establishment of healthy growth, development, and breastfeeding [5, 6]. The World Health Organization recommends immediate and exclusive breastfeeding (EBF), and summarized in 2008 a set of health conditions affecting the infant or mother that warrant alternative feeding [7, 8]. Intrauterine, postnatal, and when complementary foods are introduced are critical phases of growth during infancy. A particularly vulnerable time for growth is immediately after birth, when infants typically lose 5–9% of their birth weight prior to beginning weight gain [9, 10]. While some weight loss is normal, more pronounced weight loss may increase risk of dehydration and growth impairment. In low- and middle-income countries (LMIC), growth problems frequently begin in early infancy and can be difficult to reverse [6, 11, 12]. Preliminary data suggest that two important early predictors of infant growth impairment in sub-Saharan Africa are low birth weight (LBW) and among those not LBW, weight <2600 grams (g) at 4 days of age (data not yet published).

Optimal nutrition can prevent wasting and stunting [13–15]. EBF is widely recommended and provides optimal nutrition for most infants [8, 16], but some exclusively breastfed infants in LMIC fail to thrive. Providing additional nutrition to breastfeeding during early infancy, particularly to infants who initially fail to thrive, may have the potential to prevent wasting and stunting and improve health outcomes [4, 17]. Formula supports weight gain of at-risk infants in early infancy [9, 18, 19]. Tested in two randomized controlled trials in the United States, early, daily, small-volume formula supplementation supported growth without interfering with overall breastfeeding duration [20, 21]. In LMIC, formula may allow infants at risk of future wasting and stunting to begin weight gain earlier; however, formula use has also been associated with mortality [22–24]. Studies have shown that the impact of formula on infant weight can vary by infant age and by breastfeeding status. While formula use increases infant weight in the neonatal period, exclusive use of formula feeding decreases infant weight in the first 2 months, and then increases infant weight later in infancy [9, 18, 25]. Thus, studies are needed in LMIC to demonstrate the effect of formula feeding on infant growth and assess the frequency of adverse events [23, 24].

## Methods and analysis

### Study design and setting

**Fig 1:**
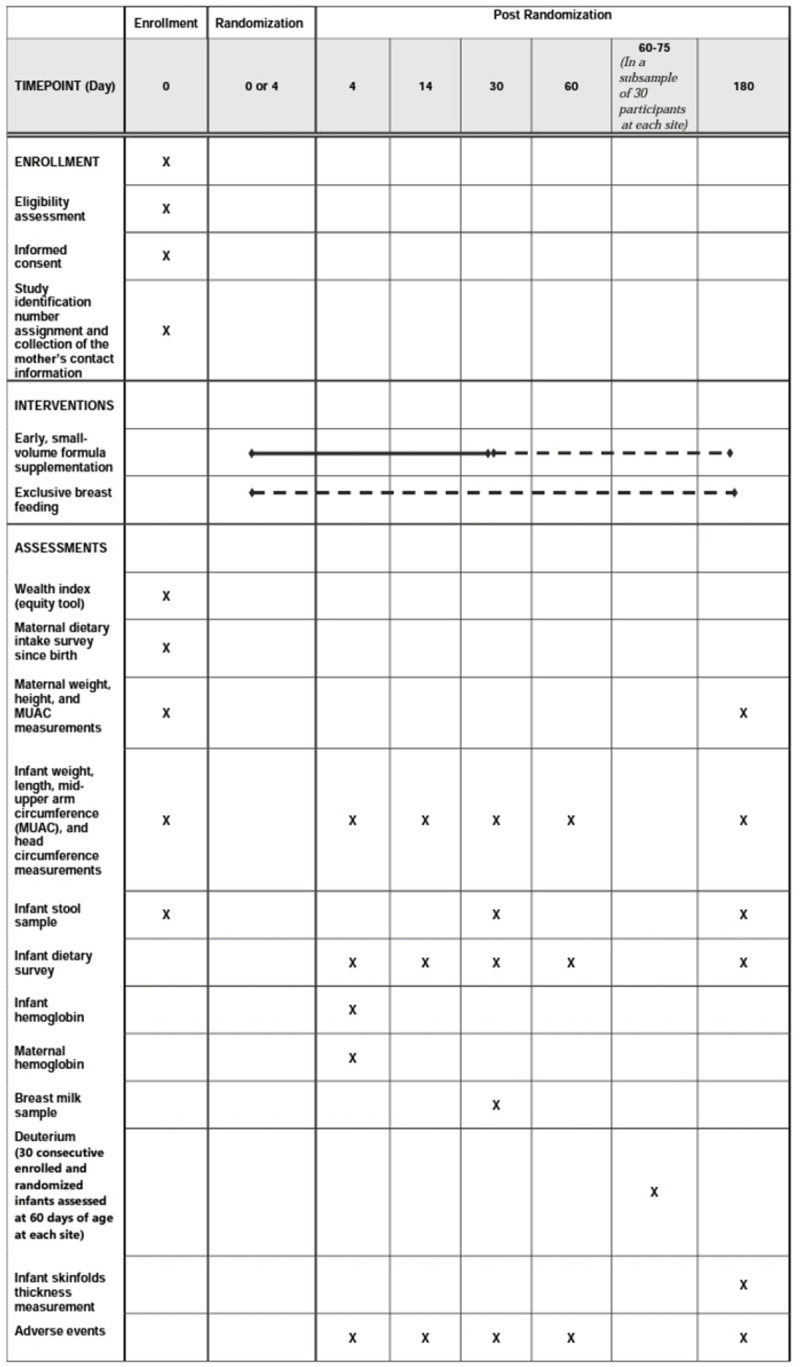
Schedule of enrolment, interventions, and assessments.

The primary objective of this study is to evaluate the efficacy of formula for increasing weight-for-age z-score (WAZ) at 30 days of age. We chose WAZ rather than weight-for-length z-score (WLZ) or length-for-age z-score (LAZ) as our primary outcome because of WAZ high measurement accuracy. We will conduct a prospective, randomized controlled trial among LBW infants and those not LBW with weights <2600 grams (g) at 4 days of age to compare breastfeeding combined with formula through 30 days of age vs recommendations for EBF without supplementation (Fig 1). We will test the hypothesis that, compared to EBF, formula increases WAZ at 30 days of age (primary outcome) among high-risk infants. Enrolled mother-infant pairs (dyads) will be followed for 6 months for additional outcomes including growth, intestinal microbiota, breastfeeding duration, infant dietary intake, and adverse events. Secondary and exploratory objectives include: 1) determining the effect of formula on WLZ at 30 days of infant age; 2) determining the effect of formula on WAZ, WLZ and LAZ through 6 months of infant age; 3) measuring the effect of formula on breastfeeding duration through 6 months of infant age; and 4) determining the effect of formula on the abundance of *Bifidobacterium infantis* in the infant intestinal microbiota at 30 days of infant age.

Beginning in February 2021 and anticipated to last approximately 12 months, this trial will be conducted at Simão Mendes and Cumura Missionary Hospitals in Bissau, Guinea-Bissau and Kawempe Hospital in Kampala, Uganda. These study settings were selected based on our preliminary qualitative data demonstrating readiness, including high levels of healthcare provider knowledge regarding the benefits of breastfeeding and the importance of avoiding unsafe breast milk substitutes such as cow’s milk [26]. Patients and/or the public were not involved in the design, or conduct, or reporting, or dissemination plans of this research. The study staff will be trained in Good Clinical Practice (GCP) as well as study-specific training according to study role(s).

### Study population

The study population will consist of mothers giving birth in participating hospitals and their singleton infants who weigh 2000-2885g at <6 hours of age. A weight maximum of 2885g was selected for the eligibility criteria because infants with birth weight <2500g will be randomized on the day of birth, whereas infants with birth weight ≥2500g will be weighed again on day 4 and will be randomized only if they weigh <2600g on day 4 and have not lost ≥10% of their birth weight. Thus, any infant weighing >2885g at birth will not be eligible for randomization in the study. Trained study staff will screen interested dyads referred by local clinicians during the birth hospitalization. Dyads will be screened in a sequential manner, as much as possible. Trained study staff will assess each dyad for all eligibility criteria (Table 1). Final eligibility for enrollment on day 0 and then randomization on days 0 or 4 will depend on the medical history and examination, appropriate understanding of the study by the mother, and completion of the written informed consent process (S1 File).

**Table 1:** Study eligibility criteria, visits, measurements, and endpoints.

**Eligibility criteria**	
Inclusion criteria for screening	• Infant < 6 hours old • Infant birth weight estimated at 1500–3500 grams (g) • Mother ≥ 18 years old • Mother intends to breastfeed • Mother with negative HIV test • Mother lives in study catchment area and anticipates availability for all study visits
Exclusion criteria for screening	• Infant a twin or multiple • Infant with known major congenital anomaly, including orofacial cleft, neural tube defect or congenital heart defect • Infant with World Health Organization (WHO) newborn or respiratory danger sign present, including lethargy or unconsciousness, no spontaneous movement, not feeding well, convulsions, raised temperature > 37.5 degrees Celsius, hyopthermia < 35.5 degrees Celsius, jaundice, very fast breathing ≥ 60 breaths/minute, severe chest indrawing, head nodding, flaring, or grunting • Contraindication to breastfeeding at each site as determined by the site’s national or sub-national health authorities • Mother with psychiatric or psychosocial barrier to study participation • Infant enrolled in another study • Mother has had another infant enrolled in this study • Mother unable or unwilling to complete all aspects of the protocol
Inclusion criteria for enrollment	• Infant < 6 hours of age with screening weight 2000–2885 g • All screening inclusion criteria met
Exclusion criteria for enrollment	• Infant < 6 hours of age with screening weight < 2000 g or > 2885 g • Any screening exclusion criteria met
Inclusion criteria for randomization at day 0	• Enrolled infant < 6 hours of age with screening weight 2000–2499 g • All screening inclusion criteria met
Exclusion criteria for randomization at day 0	• Any screening exclusion criteria met
Inclusion criteria for randomization on calendar day 4	• Enrolled infant with weight < 2600 g • All screening inclusion criteria met
Exclusion criteria for randomization on calaendar day 4	• Enrolled infant with weight loss ≥ 10% screening weight on day 4. • Infant with WHO newborn or respiratory danger sign present, including lethargy or unconsciousness, no spontaneous movement, not feeding well, convulsions, raised temperature > 37.5 degrees Celsius, hyopthermia < 35.5 degrees Celsius, jaundice, very fast breathing ≥ 60 breaths/minute, severe chest indrawing, head nodding, flaring, or grunting • Contraindication to breastfeeding at each site as determined by the site’s national or sub-national health authorities • Mother with psychiatric or psychosocial barrier to study participation • Infant enrolled in another study • Mother has had another infant enrolled in this study • Mother unable or unwilling to complete all aspects of the protocol
**Study visits**	
Day 0	• Informed consent obtained • Mother-infant identification number assigned • Infant and maternal contact information, sociodemographic characteristics, current clinical status, and medical history obtained • Infant weight, length, mid-upper arm circumference (MUAC), head circumference measured • Infant physical examination performed • Infant stool sample collected • Maternal weight, height, and MUAC measured • Randomization completed if eligible for randomization
Day 4	• Infant weight, length, MUAC, head circumference measured • Infant hemoglobin measured • Infant and household dietary survey administered • Infant adverse events recorded • Maternal hemoglobin measured • Randomization completed if not already randomized and eligible for randomization
Day 14	• Infant weight, length, MUAC, head circumference measured • Infant dietary survey administered • Infant adverse events recorded
Day 30	• Infant weight, length, MUAC, head circumference measured • Infant stool sample collected • Infant and household dietary survey administered • Infant adverse events recorded • Maternal breast milk sample collected
Day 60	• Infant weight, length, MUAC, head circumference measured • Infant and household dietary survey administered • Infant adverse events recorded • Milk transfer assessed
Day 180 (study exit)	• Infant weight, length, MUAC, head circumference, skin folds measured • Infant stool sample collected • Infant and household dietary survey administered • Infant adverse events recorded • Maternal weight, height, and MUAC measured
**Study measurements**	
Infant weight	• Two infant weights will be obtained at specified study visits, and if these duplicate weights vary by 10 g or more, an additional 2 infant weights will be obtained. Each weight that varies 10 g or less from another weight will be averaged to determine the infant weight.
Infant length	Two infant length measurements will be obtained at specified study visits, and if these duplicate lengths vary by 0.5 centimeters (cm) or more, an additional 2 infant length measurements will be obtained. Each length measurement that varies 0.5 cm or less from another length measurement will be averaged to determine the infant length.
Infant MUAC	Two infant MUAC measurements will be obtained at specified study visits, and if these duplicate MUAC vary by 0.2 cm or more, an additional 2 infant MUAC measurements will be obtained. Each MUAC measurement that varies 0.2 cm or less from another MUAC measurement will be averaged to determine the infant MUAC.
Infant skinfold thickness	Two infant skinfold thickness measurements will be obtained at the biceps and at the triceps at the specified study visit, and if these duplicate skinfold thickness measurements vary by 0.2 cm or more, an additional 2 infant skinfold thickness measurements will be obtained. Each skinfold thickness measurement that varies 0.2 cm or less from another skinfold thickness measurement will be averaged to determine the infant skinfold thickness.
Maternal weight	Two maternal weights will be obtained at specified study visits, and if these duplicate weights vary by more than 0.5 kilograms (kg), an additional 2 maternal weights will be obtained. Each weight that varies 0.5 kg or less from another weight will be averaged to determine the maternal weight.
Maternal height	Two maternal height measurements will be obtained at specified study visits, and if these duplicate heights vary by more than 0.5 centimeters (cm), an additional 2 maternal height measurements will be obtained. Each height measurement that varies 0.5 cm or less from another height measurement will be averaged to determine the maternal height.
Maternal MUAC	Two maternal MUAC measurements will be obtained at specified study visits, and if these duplicate MUAC vary by more than 0.2 cm, an additional 2 maternal MUAC measurements will be obtained. Each MUAC measurement that varies 0.2 cm or less from another MUAC measurement will be averaged to determine the maternal MUAC.
Adverse event	Any untoward medical occurrence
Serious adverse event	Any untoward medical occurrence that meets any of the following criteria: • Results in death • Is life-threatening • Requires inpatient hospitalization • Results in persistent or significant disability/incapacity • Is important AND, based upon appropriate medical judgment, may jeopardize the health of the enrolled infant or require medical or surgical intervention to prevent one of the outcomes listed above.
**Study endpoints**	
Primary endpoints	• Weight-for-age z-score at 30 days of age
Secondary and exploratory endpoints	• Weight-for-length z-score at 30 days of age • Weight-for-age z-score, weight-for-length z-score, and length-for-age z-score through 6 months of age • Duration of breastfeeding through 6 months of age • Abundance of *Bifidobacterium infantis* in the intestinal microbiota at 30 days of age

### Randomization and study procedures

Following enrollment, trained study staff will perform day 0 study procedures (Table 1) according to the most recently approved version of the protocol (current V.2.1, January 2021). Randomization to intervention group will be stratified by country and by weight at time of randomization (day 0 weight 2000–2249g; day 0 weight 2250–2499g; day 4 weight 2250–2424g; day 4 weight 2425–2599g), with 1:1 randomization within each stratum. If the enrolled infant weighs 2000–2499g on day 0, the dyad will be randomized on day 0. If the enrolled infant weighs 2500-2885g on day 0, the dyad will not be randomized on day 0; no intervention will be given on day 0 and the study team will follow the infant and weigh the infant again on day 4. If the enrolled but not yet randomized infant weighs <2600g on day 4, the dyad will be randomized on day 4; however, if the enrolled but not yet randomized infant weighs ≥2600g or has a weight loss ≥10% of birth weight on day 4, the dyad will exit the study. Random allocation will be accessed through a password-protected secure program (randomize.net) on a study device. While study staff and mothers will not be blinded to intervention assignment, the study staff will have no foreknowledge of intervention assignment. Participants will be enrolled with complete concealment of intervention so that both study staff and participants will learn of intervention allocation upon randomization.

Various mitigation measures will be put in place to reduce the possibility that the study’s conduct will impact the acceptability or distribution of formula within the postpartum ward or healthcare provider counselling and support for breastfeeding. First, all formula will be kept out of sight of patients and healthcare providers in a locked cabinet or locked room unless in active use by study staff or participants. Second, study activities will occur in a private room within in the hospital or in the participant’s home rather than on the postpartum ward. Third, prior to randomization, formula will be stored in an opaque container, so that only mothers who are randomly assigned to the study intervention will see it. Fourth, the study will not advertise for participants and will not distribute any materials to participants other than those required by the respective ethics committees.

For enrolled dyads who are randomized, the intervention will either be 1) breastfeeding combined with Similac Pro-Advance formula consisting of up to 59 milliliters (mL) of individually-prepackaged, single-use liquid, iron-fortified infant formula (Similac, Abbott Nutrition, Columbus, Ohio) administered daily through 30 days of infant age followed by EBF through 6 months of infant age; or 2) frequent EBF without additional food or fluid (including water) through 6 months of infant age (control, current standard of care). According to available product information, the intervention formula contains 64.3 kcal/100 mL with a protein content of 1.33 g/100mL and iron content of 1.2 mg/dL.

For dyads randomly assigned to the formula intervention, trained study staff will instruct the mother to breastfeed the infant on demand and at least 8 times a day, and to supplement the infant daily by offering a single-use 59-mL bottle of formula immediately following a breastfeeding and allowing the infant to consume until the infant discontinues feeding and within one hour of opening. The amount of formula consumed by the infant at each feeding will be recorded by study staff. A single-use sterile nipple will also be provided with each bottle. Trained study staff will directly observe four daily formula feedings and then subsequently will deliver two 59 mL formula bottles and two single-use sterile nipples to mothers every other day until 30 days of age with instructions to continue once daily formula supplementation, saving any formula leftover after the feedings to return to the study team to measure and destroy. For dyads randomly assigned to EBF, trained study staff will provide the mothers the identical breastfeeding advice as the formula group with the exception that no supplementation should be given. Medical management of participants will be provided by their usual healthcare providers, not by study staff.

On days 4, 14, 30, 60 and 180, enrolled and randomized dyads will be assessed by trained study staff (Table 1). Infant (naked) weight will be measured using a Seca 334 scale (Seca Inc., Wandsbek, Germany) calibrated before each use. Infant length will be measured using a Seca 416 infant stadiometer and infant mid-upper arm circumference (MUAC) measurement using infant MUAC tape. When measuring infant skinfold thickness, standard skinfold calipers will be used. Maternal weight, height, and MUAC will be measured using standard scales, stadiometers, and MUAC tape, respectively. Infant and maternal hemoglobin will be measured by Hemocue at the day 4 study visit and infant stool samples collected for future intestinal microbiota analysis at the days 0, 30 and 180 study visits. An infant dietary survey (S1 File) will be administered at each scheduled study visit. All infants will be monitored at each study visit for signs or symptoms of illness (fever, low body temperature, blood in stool, profuse/watery diarrhea, vomiting, difficulty breathing, lethargy/decreased consciousness, convulsions, jaundice, and umbilical redness) or any growth problems or adverse events, including serious adverse events. If infant death is ascertained at any follow-up study visit, trained study staff will administer the relevant WHO verbal autopsy instrument.

Maternal milk samples will be obtained from consenting enrolled mothers on the day 30 study visit immediately following breastfeeding, with mothers asked to hand express 10 mL breast milk from each breast for a total of 20 ml. These breast milk samples will be frozen for future analysis. An infant breast milk volume measurement at 2 months is planned in a subsample of 60 mothers, 30 infants at each study site, by specially trained study staff using the deuterium oxide dose-to-the-mother method, which involves mothers consuming a small volume of deuterium oxide diluted in a beverage and collecting subsequent maternal and infant urine samples over a series of days.

### Sample size

In each country, trained study staff will weigh up to 1,064 infants to identify and randomize 72 with birth weight ≤2500g and 90 who weigh <2600g on day 4. With respect to the primary aim, determining the impact of formula on WAZ at 30 days of infant age, the anticipated sample size of 324 dyads (144 LBW infants and 180 infants with low weight on day 4 under equal randomization) will provide 80% or greater power per weight stratum for intervention group comparison to detect a difference of 0.20 with respect to the outcome of WAZ at day 30 for the entire study cohort after accounting for up to 20% loss to follow-up. This sample size will also allow the estimation of the mean difference between WAZ at day 30 and WAZ at birth with 0.17 and 0.15 standard errors by intervention group, respectively, for the entire study cohort. Including both mothers and infants, the total sample size for the randomized trial is anticipated to be 324 mothers and 324 infants, or 648 individual participants in total.

### Data collection and quality assurance

Study data will be collected using standardized electronic data instruments and entered directly into study databases during study visits by trained study staff. Paper case report forms (pCRFs) will be used when electronic data collection is not feasible, and paper forms will be used to document informed consent, participant location information, adverse events, and protocol noncompliances. Any data obtained on pCRFs will be electronically entered into the study database as soon as possible and at least weekly. Data collected electronically will be uploaded to the secure study database at least weekly in a de-identified manner to a secure, password-protected server and extracted for statistical analyses. Participant information with personal identifiers will be stored in paper-based form in a locked filing cabinet in a secure and locked private office or on a password-protected secure server. To ensure accuracy and completeness, study data will be routinely reviewed by study site staff trained in quality control and assurance as well as through both remote monitoring and study site visits by the co-investigators who will conduct data quality assurance reviews, audits, and evaluation of the study safety and progress. Standard GCP will be followed to ensure accurate, reliable, and consistent data collection.

### Data management

Study data management activities including data entry and validation, data coding and cleaning, database quality control, adverse event reporting and tracking systems, preparation and submission of safety and compliance reports, and preparation of final study database will be overseen at the respective study sites in collaboration with University of California, San Francisco (UCSF) data management staff using Research Electronic Data Capture (REDCap) software. The study site teams will be responsible for maintaining, and storing securely, complete, accurate and current study records throughout the study including signed informed consent. Data queries will be resolved weekly, and missing data accounted for in statistical analyses. All data management activities will be in compliance with International Council on Harmonization (ICH) GCP E6, locally applicable institutional review board, regulatory, sponsoring organization, and institutional requirements for the protection of study participants and confidentiality of personal and health information. After publication of the primary results, the data underlying the findings will be made available in a self-service, online public data repository.

### Outcomes

The primary study outcome is infant WAZ at 30 days of infant age. We hypothesize that feeding formula to infants will increase infant WAZ at 30 days of infant age by 0.20 compared to infants randomly assigned to recommendations for EBF without supplementation. Secondary, exploratory, and safety study outcomes include infant WLZ at 30 days of infant age; WAZ, WLZ, and LAZ through 6 months of age; breastfeeding duration; abundance of *B*. *infantis* in the intestinal microbiota at 30 days of age; adverse events; and serious adverse events. We hypothesize that feeding formula will increase infant WLZ at 30 days of infant age and infant WAZ, WLZ, and LAZ through 6 months of age by 0.20 compared to infants randomly assigned to recommendations for EBF without supplementation. While we hypothesize that infant breastfeeding duration will not differ between these feeding interventions, we hypothesize that the abundance of *B*. *infantis* in the intestinal microbiota will be higher among infants at 30 days of age who receive formula than among those who do not. To ensure infant safety, the effect of formula on mortality will be assessed after every 20 randomized infants have completed 30 days of follow-up, and the effect of formula on mortality and SAEs will be assessed once 25% and once 50% of randomized participants have completed 30 days of follow-up.

### Statistical analyses

Our primary analysis will be intention-to-treat which will inform the primary global health policy question of whether recommending formula improves infant growth. In order to account for our planned stratified randomization, we will use a permutation test, permuting the infant feeding intervention assignment within the infant weight stratum, to determine the effect of formula on the primary outcome of WAZ at 30 days of infant age as well as on the secondary and exploratory outcomes of WLZ at 30 days, WAZ, WLZ and LAZ through 6 months, intestinal microbiota at 30 days of age, breastfeeding duration, and receipt of non-breast milk dietary intake between birth and 6 months of age.

To determine *B*. *infantis* abundance, stool samples will be shipped to University of California, Davis for analysis and assessed by 16S rRNA gene sequencing. Samples found to have sequences from the *Bifidobacteriaceae* family will be further subjected to analysis of bifidobacterial species-specific terminal restriction fragment length polymorphisms (Bif-TRFLP) to identify specific species of *Bifidobacterium*. Statistical differences in *B*. *infantis* abundance between supplemented and unsupplemented infants, while accounting for strata, will be assessed by mixed linear modeling. In additional exploratory analyses, we will use cluster and eigenstructure analyses (e.g., principal component analysis) for descriptive analysis of 16S rRNA gene abundance data, followed by adonis permutation tests for significance of associations with continuous or factor variables or Mantel tests for associations between distance matrices. For example, feeding intervention group is an important factor variable; a significant association of the 16S rRNA gene profile with group membership would suggest an effect of formula on microbiota. Additional exploratory analyses will be undertaken with supervised machine-learning approaches, in order to identify new and unexpected features unique to the intestinal environment of formula recipients. For example, random forests will be used to identify important features separating the microbiotas of formula from EBF recipients.

## Ethics and dissemination

### Ethical approvals and consent

The study will be conducted in accordance with the ICH GCP and the Declaration of Helsinki 2008. The protocol and other relevant study documents were approved by the UCSF Institutional Review Board (19–29405); the Guinea-Bissau National Committee on Ethics in Health (Comite Nacional de Etica na Saude, 075/CNES/INASA/2020); the Higher Degrees, Research and Ethics Committee of Makerere University (871); and the Uganda National Council of Science and Technology (HS1226ES). Local clinicians will inform mothers about the study and refer those who are interested to study staff. Trained study staff will review the birth record to confirm eligibility for screening and then initiate contact with the mothers. Written informed consent will be obtained in the local language by trained study staff from eligible study participants prior to enrollment. Potential participants will have adequate time to ask questions and a comprehension checklist will be administered to ensure participant understanding.

To address the risk that the conduct of this study could result in changes to current infant feeding recommendations or practices at the local hospitals or any violation to the International Code of Marketing of Breast-milk Substitutes, targeted mitigation measures will be undertaken. First, the importance of frequent, on-demand breastfeeding will be emphasized to all study participants. Second, the study will not advertise for recruitment, but instead will rely on referrals from hospital staff who are educated about the benefits of breastfeeding. Third, informed consent will provide all potential participants with information about the risks of formula supplementation. Fourth, all formula will remain securely stored in a locked storage unit unless in active use. Fifth, formula use will be demonstrated only to mothers in the small-volume formula supplementation group, and formula bottles will remain concealed in an opaque container until randomization occurs. Any protocol violations will be reported to the appropriate ethics committees.

### Safety

There will be close safety monitoring of all enrolled and randomized dyads. Each participating infant will be evaluated by a study clinician at each study visit and if necessary, referred to local healthcare providers for management. Stringent COVID-19 precautions to mitigate all potential exposures will be taken throughout the study. Visits will be conducted either at the participant’s home or at the study site to ensure timely assessment. Every effort will be made to trace all dyads in the study for the final outcome assessment. An emergency number will be provided to all study participants so that an on-call clinician can be reached at any time during study participation. As needed, infants in the study will be seen at interim visits and referral facilitated for those with concerning signs or symptoms or measured weight loss ≥10% of birth weight or for any other necessary care. All adverse events will be assessed and managed in accordance with standard clinical practice at the study sites and documented by the study team. Infants with an adverse event will be followed and treated until the adverse event is resolved or stabilized. All serious adverse events occurring after randomization will be reported to the principal investigator and a study safety medical officer for review within 24 hours of the event. Serious adverse events occurring after randomization will be regularly reviewed by the study medical monitor and compiled into reports for the co-investigators. An independent 5-person data safety and monitoring board (DSMB) will review cumulative safety and study conduct data through two formal interim analyses. At a minimum, safety data presented to the DSMB will include summaries of data on adverse events, serious adverse events, and adherence rates among randomized infants. The DSMB will consider recommending to stop the trial prior to maximum enrollment if they determine there are safety concerns.

### Possible risks

There are potential risks to study participation. This is a randomized trial that is investigating the efficacy of formula compared to EBF, and it is possible that formula may increase the risk of diarrhea, infectious diseases, and breastfeeding cessation, exposing infants in the formula group to increased risk of adverse events, hospitalization or death compared to infants in the EBF group. It is also possible that formula avoids underweight wasting and stunting in early infancy and that infants in the EBF group could experience higher rates of growth failure, with an increased risk of adverse events, hospitalization, or death. Diarrhea, infectious diseases, breastfeeding cessation, and growth failure are common risks in infancy in LMIC, and both breastfeeding with supplementation and EBF are common. For this reason, the risks to infant study participants are not anticipated to differ from the ordinary risks of infancy. The purpose of this study is to determine if the incidence of any of these risks differs between the feeding groups, with the hypothesis being that these metrics are improved in the formula group compared to the EBF group. Furthermore, the study was carefully developed and pragmatically designed with inclusion and exclusion criteria to allow results as generalizable as possible without putting infants with severe illness at risk and a robust safety monitoring scheme will be in place. Infants most at risk of adverse consequences from formula will be excluded from this study, including those with birth weight <2000g, those with weight loss ≥10% on day 4, those with congenital abnormalities, and those with WHO danger signs. To further ensure safety, if any medical or safety issues are identified, study staff will confirm that study participants have been appropriately and expeditiously referred to local healthcare providers for management.

Recognizing that some study participants may not come back for the follow-up visits, trained study staff will attempt to locate infants who miss their follow-up appointments and conduct these visits in the home. We will also ensure that study staff take the time to educate mothers on the importance of breastfeeding, adhering to the assigned intervention, and follow-up.

### Dissemination

We plan to disseminate study results in peer-reviewed journals and international conferences, targeting those involved in the clinical care of infants in low-resource settings as well as those who develop and advise on policies and guidelines in those settings. The trial is registered with ClinicalTrials.gov (registration number NCT04704076).

### Efforts towards a rigorous protocol

Dedicated study staff trained in GCP and study-specific procedures will follow enrolled dyads to assure the protocol and standard operating procedures are followed and data are accurately collected. Standardized study-specific training, supervision, and oversight will be undertaken to ensure quality, consistency, and harmonized trial procedures and implementation. Regular monitoring will be provided by the co-investigators to assess compliance with human subjects and other research regulations and guidelines, adherence to the study protocol and procedures, and quality and accuracy of data collected.

### Limitations and bias

In addition to the inherent limitations of any study with a limited sample size and strategy, limitations to this study and potential sources of bias include infant feeding intervention non-adherence and loss to follow-up. A major study consideration is that some infants randomly assigned to the recommendation of EBF will receive some form of supplementation, a potential difference between the randomized groups that will also be measured with the deuterium oxide dose-to-the-mother method as described above. The premise of our study is that carefully managed supplementation with formula may benefit infant growth when compared to a recommendation of EBF that may result in either EBF or in unstructured supplementation with liquids of low nutritional value. The purpose of this is to compare formula with the current standard of care of recommending EBF, with the understanding that the EBF recommendation will be followed by some and not by others. To minimize non-adherence for those randomized to the EBF group, EBF counseling will be provided at each study visit as well as an infant dietary survey. To minimize non-adherence for those randomized to the formula group, breastfeeding counseling, hands-on training, and demonstrations of formula administration will be conducted. Mothers will be provided with clear and consistent instructions and initial direct observation of mothers providing formula to their infant will be conducted. No more than two bottles of formula will be delivered to participants at any one time. Any unused formula as well as used bottles and nipples will be collected each time formula is delivered, and an infant dietary survey will be administered at each study visit. To minimize loss to follow-up, mothers will be provided with clear follow-up instructions as well as called the afternoon before their visits when feasible to remind them of the visit the following day. A travel stipend will be provided for visits occurring at the study site. Home visits will be scheduled when preferred by the mother or upon a missed visit. Because of the frequent formula delivery to participants in the formula group and these visits do not occur in the EBF group, we may expect more adverse events reported in the formula group.

Bias also may be introduced from the increased monitoring of infant weight which will allow prompt referral of infant growth problems. Because study participants will receive extra instruction in breastfeeding recommendations as well as additional weight monitoring, study infants are anticipated to be better nourished than infants who do not participate, irrespective of random feeding assignment.

## Supporting information

S1 Checklist SPIRIT 2013 checklist: Recommended items to address in a clinical trial protocol and related documents*. (PDF)

S1 File Protocol; Uganda screening consent form; Uganda enrolment consent form; Guinea-Bissau screening and enrolment consent form; and Infant dietary survey. (DOCX)
