# Supplementary material for: Randomized controlled trial of early, small-volume formula supplementation among newborns: A study protocol
Source: PLoS One. 2022 Feb 4;17(2):e0263129. doi: 10.1371/journal.pone.0263129 (PMC8815972; doi:10.1371/journal.pone.0263129)
Supplement: S1 File — (DOCX) [file pone.0263129.s002.docx]

**Supplementary File S1** **Protocol; Uganda screening consent form; Uganda enrolment consent form; Guinea-Bissau screening and enrolment consent form; and Infant dietary survey**

1. **Protocol**

# Title: Pilot Randomized Controlled Trial: Preventing Infant Malnutrition with Early Supplementation (PRIMES)

**Sponsored by: University of California, San Francisco Funded by:**

**Bill & Melinda Gates Foundation Version 2.2**

**April 8, 2021**

**Please note: Italicized text used throughout to denote activities to be undertaken if circumstances such as the COVID-19 pandemic permit.**

Contents

ABBREVIATIONS AND ACRONYMS [5](#_bookmark0)

PRINCIPAL INVESTIGATOR [6](#_bookmark1)

CO-INVESTIGATORS [6](#_bookmark2)

EXECUTIVE SUMMARY [7](#_bookmark3)

BACKGROUND AND RATIONALE [8](#_bookmark4)

HYPOTHESES AND OBJECTIVES [9](#_bookmark5)

SPECIFIC AIMS [9](#_bookmark6)

HYPOTHESES [10](#_bookmark7)

METHODOLOGY [10](#_bookmark8)

DESIGN [10](#_bookmark9)

SITES [10](#_bookmark10)

POPULATION [11](#_bookmark11)

INCLUSION CRITERIA FOR SCREENING: [11](#_bookmark12)

EXCLUSION CRITERIA FOR SCREENING: [11](#_bookmark13)

CRITERIA FOR INCLUSION IN STUDY: [12](#_bookmark14)

CRITERIA FOR INCLUSION IN RANDOMIZATION ON DAY 0 (AT ENROLLMENT):

[................................................................................................................................................12](#_bookmark15)

CRITERIA FOR EXCLUSION FROM RANDOMIZATION ON DAY 0 (AT ENROLLMENT): [12](#_bookmark16)

CRITERIA FOR INCLUSION IN RANDOMIZATION ON DAY 4: [12](#_bookmark17)

CRITERIA FOR EXCLUSION FROM RANDOMIZATION ON DAY 4,: [12](#_bookmark18)

STUDY PERIOD [13](#_bookmark19)

SAMPLE SIZE [13](#_bookmark20)

RECRUITMENT [14](#_bookmark21)

RANDOMIZATION [14](#_bookmark22)

CLINICAL MANAGEMENT OF STUDY PARTICIPANTS [15](#_bookmark23)

STUDY VISITS [15](#_bookmark24)

STUDY TERMINATION VISIT [16](#_bookmark25)

MISSED VISITS [16](#_bookmark26)

STUDY PROCEDURES [16](#_bookmark27)

CALENDAR OF INFANT ASSESSMENTS [18](#_bookmark28)

CALENDAR OF MATERNAL ASSESSMENTS [19](#_bookmark29)

TREATMENT ASSIGNMENT TO INTERVENTION OR CONTROL [20](#_bookmark30)

DATA COLLECTION [20](#_bookmark31)

TRAINING [21](#_bookmark32)

ADVERSE EVENTS [21](#_bookmark33)

Serious Adverse Events [21](#_bookmark34)

Serious Adverse Event Relationship to ESVS [21](#_bookmark35)

SAFETY REPORTING [22](#_bookmark36)

STUDY DISCONTINUATION [22](#_bookmark37)

DATA MANAGEMENT [22](#_bookmark38)

STUDY MONITORING [23](#_bookmark39)

DATA STORAGE [23](#_bookmark40)

STATISTICAL ANALYSIS [24](#_bookmark41)

DATA SAFETY AND MONITORING PLAN (DSMP) [24](#_bookmark42)

DATA SAFETY AND MONITORING BOARD [25](#_bookmark43)

RESULTS PRESENTATION AND DISSEMINATION [25](#_bookmark44)

ETHICAL CONSIDERATION AND CONSENT [26](#_bookmark45)

INSTITUTIONAL REVIEW BOARDS (IRBs) AND INDEPENDENT ETHICS COMMITTEES (IECs). [26](#_bookmark46)

INFORMED CONSENT [26](#_bookmark47)

RISKS TO PARTICIPANTS [27](#_bookmark48)

PROTECTION AGAINST RISKS [27](#_bookmark49)

BENEFITS TO PARTICIPANTS [29](#_bookmark50)

PARTICIPANT CONFIDENTIALITY [29](#_bookmark51)

REIMBURSEMENT [29](#_bookmark52)

POSSIBLE CONSTRAINTS [29](#_bookmark53)

WITHDRAWAL AND EARLY TERMINATION [30](#_bookmark54)

PARTICIPANT RETENTION [30](#_bookmark55)

BIOHAZARD CONTAINMENT [30](#_bookmark56)

PERSONNEL [31](#_bookmark57)

SUPPLIES [31](#_bookmark58)

BUDGET [31](#_bookmark59)

REFERENCES [33](#_bookmark60)

APPENDIX [35](#_bookmark61)

APPENDIX: SCHEDULE OF STUDY VISITS [35](#_bookmark62)

# ABBREVIATIONS AND ACRONYMS

AE: adverse event

BMGF: Bill & Melinda Gates Foundation COVID-19: Coronavirus Disease 2019 DOB: date of birth

DSMB: Data safety and monitoring board EBF: exclusive breastfeeding

ESVS: early small volume supplementation GCP: Good Clinical Practice

ID: identification

IRB: institutional review board LAZ: length/height-for-age z-score LBW: low birth weight

LMIC: low- and middle-income countries

MakSPH: Makerere University School of Public Health MUAC: mid-upper arm circumference

PI: Principal Investigator

PRIMES: Preventing Infant Malnutrition with Early Supplementation REDCap: Research Electronic Data Capture

SAE: serious adverse event SE: standard error

SOP: standard operating procedure TOB: time of birth

UCSF: University of California, San Francisco UNICEF: United Nations Children’s Fund WAZ: weight-for-age z-score

WHO: World Health Organization WLZ: weight-for-length z-score

# PRINCIPAL INVESTIGATOR

Valerie Flaherman, MD, MPH

Professor of Pediatrics and Epidemiology and Biostatistics University of California, San Francisco

Email: [Valerie.flaherman@ucsf.edu](mailto:Valerie.flaherman@ucsf.edu) Phone: (414) 502-6266

Address: 3333 California Street, 245 San Francisco, CA 94118

# CO-INVESTIGATORS

Susan Roberts, PhD Consultant to this project, and

Professor of Nutrition, Tufts University Email: [susan.roberts@tufts.edu](mailto:susan.roberts@tufts.edu)

Augusto Braima De Sa

International Partnership for Human Development Email: [saaugusto28@hotmail.com](mailto:saaugusto28@hotmail.com)

Victoria Nankabirwa, MBBS, PhD Lecturer, Makerere University Emai[l: nankabirwav@gmail.com](mailto:nankabirwav@gmail.com)

Olive Jackie, MBBS, PhD (c) Research Fellow, Makerere University [olivedejackie@yahoo.com](mailto:olivedejackie@yahoo.com)

Dr. Jolly Nankunda, MBChB, MMed , PhD,

Senior Consultant Pediatrician and Deputy Director Mulago Women and Neonatal Specialized Hospital [jnankunda@gmail.com](mailto:jnankunda@gmail.com)

Amy Sarah Ginsburg, MD, MPH IMG Consulting LLC

Emai[l: messageforamy@gmail.com](mailto:messageforamy@gmail.com)

Nadia Diamond-Smith, PhD, MS

Assistant Professor of Epidemiology & Biostatistics University of California, San Francisco, School of Medicine Email: [Nadia.Diamond-Sm](mailto:Nadia.Diamond-Smith@ucsf.edu)[ith@ucsf.edu](mailto:ith@ucsf.edu)

Mijung Park, PhD, MPH, RN

Assistant Professor of Family Health Care Nursing University of California, San Francisco, School of Nursing Email: [Mijung.Park@ucsf.edu](mailto:Mijung.Park@ucsf.edu)

Mi-Ok Kim, PhD

Professor of Epidemiology and Biostatistics

University of California, San Francisco, School of Medicine Email : [Miok.Kim@ucsf.edu](mailto:Miok.Kim@ucsf.edu)

Victoria Laleau, MS

University of California, San Francisco Email: [Victoria.laleau@ucsf.edu](mailto:Victoria.laleau@ucsf.edu)

# EXECUTIVE SUMMARY

**Background:** Worldwide, more than 50 million children under 5 years of age demonstrate wasting with a weight-for-length/height z-score (WLZ) <-2, and over 150 million demonstrate stunting with a length/height-for-age z-score (LAZ) <-2.1 Such wasting and stunting often begin during infancy.1 Optimal nutrition can prevent wasting and stunting.2 Exclusive breastfeeding (EBF) is widely recommended and provides optimal nutrition for most infants.3-5 However, early growth faltering is common for infants in low and middle income countries (LMIC) and can both increase an infant’s risk of early mortality and also lead to deficits in attained height and weight throughout childhood.6,7,8 Thus research is needed to determine the most efficacious strategy to promote healthy early growth in LMIC.

**Objective:** The proposed study will test the efficacy of early small volume supplementation (ESVS) for increasing weight-for-age z-score (WAZ) at 30 days of age.

**Methodology:** The PRIMES pilot will be a randomized controlled clinical trial enrolling infants weighing ≥2000g at birth in Guinea-Bissau and Uganda to different feeding groups. Infants weighing 2000-2499g at <6 hours of age (n=144; 72 per site) will be randomized on enrollment to one of two groups: 1) frequent breastfeeding combined with ESVS consisting of up to 59-mL formula administered daily through 30 days of age (intervention) followed by EBF through 6 months of age; or 2) frequent EBF without additional food or fluid (including water) other than vitamins, minerals and medications (control) through 6 months of age. Infants weighing 2500- 2885g at <6 hours of age will be weighed again at 4 days of age; those weighing <2600g at 4 days of age (n=180; 90 per site) will be randomized to the same intervention and control groups. Weight will be measured on all enrolled infants at birth or Day 1 and at Days 4, 14, 30, 60 and

180. Additional measures including height, mid-upper arm circumference (MUAC), skinfolds, *and hemoglobin* will be assessed at other time points. The study’s primary outcome will be WAZ at 30 days of age. Secondary outcomes will include WLZ at 30 days of age; WAZ, WLZ and LAZ through 6 months of age; neurodevelopmental outcomes breastfeeding duration; and *infant intestinal microbiota*.

# BACKGROUND AND RATIONALE

Childhood undernutrition is a major health burden worldwide that increases childhood morbidity and mortality and causes problems in infant development that can persist into adulthood.6 Although some countries have achieved substantial progress in preventing or treating childhood malnutrition in the past two decades, others have not. In 2016, it was estimated that globally, 25% of children under 5 are stunted due to malnutrition, with the greatest burden of disease in Africa and Asia.1 For instance, of all children with wasting (WLZ <-2) under 5 years of age, 69% were in Asia and 27% in Africa. Of all children with stunting (LAZ <-2), under 5 years of age, 56% were in Asia and 38% in Africa.

Wasting and stunting have immense worldwide repercussions for morbidity and mortality in childhood and have been associated with a range of adverse health effects.6,9,10 In the short-term, children with inadequate growth have lower survival rates and high mortality from pneumonia, diarrhea and other infectious diseases.8 The long-term effects of infant malnutrition include small stature and low body mass in adulthood, , immune dysfunction and increased risk of infectious disease8,10-12 In addition, malnutrition has been associated with lower mean total intracranial, whitematter and grey matter brain volumes, and these conditions predispose children to delays in neurodevelopment that have a profound impact on cognitive performance and on executive function. Unfortunately, in LMIC, growth problems frequently begin in early infancy and can be difficult to reverse.6,7,13 Recent evidence suggests that wasting and stunting manifested during early infancy for about 10% of those who have been breastfeeding exclusively can persist through age 5 and have a deleterious impact on childhood mortality.13

Providing additional nutrition during early infancy to those in LMIC who initially fail to thrive might prevent wasting and stunting and permit achievement of normal growth milestones by age

1. A particularly vulnerable time for growth during early infancy is immediately after birth, when exclusively breastfed infants typically lose 5-9% of their birth weight prior to beginning weight gain. In the United States, formula has demonstrated effectiveness for supporting weight gain of at-risk infants in this vulnerable period.14 Tested in two randomized controlled trials in the United States, ESVS may support growth without interfering with overall breastfeeding duration.15,16 In LMIC, ESVS might allow infants at risk of future wasting and stunting to return to birth weight earlier and thus begin weight gain earlier, but studies are needed in these countries to demonstrate effectiveness and assess the frequency of adverse events, because supplementation of breastfeeding with formula has been associated with diarrheal and other infectious diseases.17,18

This pilot study is called PReventing Infant Malnutrition with Early Supplementation (PRIMES), and parallel studies are planned to occur in Guinea-Bissau and Uganda, where there is established expertise to conduct research in infant nutrition. The long-term objective of PRIMES will be to determine whether ESVS can reduce wasting, stunting and neurodevelopmental impairment ininfancy and childhood.

PRIMES preliminary data suggests that two important risk factors for inadequate infant growth in sub-Saharan Africa are LBW (risk ratio (RR) 16.0 (5.6, 45.7) for the outcome of WAZ <-2) and among those not LBW, weight <2600g at 4 days of age (RR 15.0 (3.4, 66.5)). This pilot study will be a randomized controlled trial among LBW infants and those not LBW with weights

<2600g at 4 days of age, and will compare ESVS with recommendations for EBF to test the hypothesis that ESVS increases WAZ at 30 days of age and improves health and neurodevelopment in high-risk infants compared with recommendations for EBF without supplementation.

# HYPOTHESES AND OBJECTIVES

SPECIFIC AIMS

Primary Aim:

To determine the effect of ESVS on WAZ at 30 days of age. Secondary and Exploratory Aims:

- 1. To determine the effect of ESVS on WLZ at 30 days of age.
  2. To determine the effect of ESVS on WAZ, WLZ, and LAZ through 6 months of age.
  3. To measure the effect of ESVS on breastfeeding duration through 6 months of age.
  4. To determine the effect of ESVS on intestinal microbiota at 30 days of age.
  5. To determine the effect of ESVS on total white matter volume through 12 months of age.
  6. To determine the effect of ESVS on total brain volume through 12 months of age.
  7. To determine the effect of ESVS on neurodevelopment as measured by the Bayley Scales of Infant and Toddler Development through 12 months of age.

HYPOTHESES

We hypothesize that ESVS will increase WAZ at 30 days of age by 0.20 compared to controls randomly assigned to recommendations for EBF without supplementation.

We hypothesize that ESVS will increase WLZ at 30 days of age by 0.20 compared to controls randomly assigned to recommendations for EBF without supplementation.

We hypothesize that ESVS will increase WAZ, WLZ and LAZ through 6 months of age by 0.20 compared to controls randomly assigned to recommendations for EBF without supplementation.

We hypothesize that breastfeeding duration will not differ between ESVS and control infants.

We hypothesize that the abundance of *Bifidobacterium infantis* in the intestinal microbiota at 30 days will be higher among infants who receive ESVS than among controls.

We hypothesize that the abundance of *B.infantis* in the intestinal microbiota will be higher among infants who receive ESVS than among controls.

We hypothesize that ESVS will increase total white matter volume through 12 months of age. We hypothesize that ESVS will increase total brain volume through 12 months of age.

We hypothesize that ESVS will increase scores on the Bayley Scales of Infant and Toddler Development through 12 months of age.

# METHODOLOGY

DESIGN

Our study design is a randomized controlled clinical trial. In each country, we will weigh up to 1,064 infants to identify 72 with birth weight ≤2500g and 90 who weigh <2600g on Day 4.

Enrolled infants will be randomly assigned either to receive recommendations for frequent EBF without additional food or fluid (including water) other than vitamins, minerals and medications for 6 months (control) or to frequent breastfeeding combined with ESVS for one month (intervention). Enrolled infants will be followed through 6 months of age for outcomes including growth, *intestinal microbiota*, breastfeeding duration, non-breastfeeding dietary intake and adverse events (AEs); a subset of 80 infants will be followed through 12 months of age for these outcomes as well as neurodevelopment.

SITES

The PRIMES study will be conducted in the following locations:

1. Bissau in Guinea-Bissau; and
2. Kampala and Mukono districts, Uganda.

POPULATION

The study population will consist of singleton infants delivered in hospitals and medical centers and weighed by trained study staff at <6 hours of age with birth weight ≥2000g.

A physical exam will be conducted on all potential participants as part of the assessment for eligibility in the PRIMES study.

INCLUSION CRITERIA FOR SCREENING:

Infant < 6 hours old

Infant birth weight estimated at 1500-3500g Mother intends to breastfeed

Mother with negative HIV test

Mother lives in study catchment area and anticipates availability for all study visits Mother ≥18 years old

EXCLUSION CRITERIA FOR SCREENING:

Twins and other multiples

Infant with known major congenital anomalies including orofacial clefts, neural tube defects or congenital heart defects

Infant with WHO newborn and respiratory danger signs present:

- - Not feeding well
  - Convulsions
  - Very fast breathing ≥60 breaths/minute
  - Severe chest indrawing
  - No spontaneous movement
  - Lethargic or unconscious
  - Raised temperature > 37.5 degrees Celsius
  - Hypothermia <35.5 degrees Celsius
  - Any jaundice in first 24 hours of life or yellow palms or soles at any age
  - Head nodding, nasal flaring or grunting

Maternal psychiatric or psychosocial barrier to enrollment

Contraindication to breastfeeding at each site as determined by a site’s national or sub-national health authorities

Mothers unable or unwilling to complete all aspects of the protocol Infant enrolled in another study

Mother has had another infant enrolled in PRIMES

CRITERIA FOR INCLUSION IN STUDY:

Infant with screening birth weight at <6 hours of age 2000-2885g All screening inclusion criteria met

CRITERIA FOR EXCLUSION FROM STUDY:

Infant with screening birth weight at <6 hours of age either <2000g or >2885g No screening exclusion criteria met

CRITERIA FOR INCLUSION IN RANDOMIZATION ON DAY 0 (AT ENROLLMENT):

Infant with birth weight at <6 hours of age 2000-2499g All screening inclusion criteria met.

CRITERIA FOR EXCLUSION FROM RANDOMIZATION ON DAY 0 (AT ENROLLMENT):

Any screening exclusion criteria met.

CRITERIA FOR INCLUSION IN RANDOMIZATION ON DAY 4:

Weight <2600g

All screening inclusion criteria met

CRITERIA FOR EXCLUSION FROM RANDOMIZATION ON DAY 4,:

Infant with weight loss ≥10% birth weight on Day 4. In this case, infants will be referred to the local hospital for evaluation.

Infant with WHO newborn and respiratory danger signs present:

- - Not feeding well
  - Convulsions
  - Very fast breathing ≥60 breaths/minute
  - Severe chest indrawing
  - No spontaneous movement
  - Lethargic or unconscious
  - Raised temperature > 37.5 degrees Celsius
  - Hypothermia <35.5 degrees Celsius
  - Any jaundice in first 24 hours of life or yellow palms or soles at any age
  - Head nodding, nasal flaring or grunting

Maternal psychiatric or psychosocial barrier to enrollment

Contraindication to breastfeeding at each site as determined by a site’s national or sub-national health authorities

Mothers unable or unwilling to complete all aspects of the protocol Infant enrolled in another study

STUDY PERIOD

*We estimate that the study period will be January 2021 through December 2022*. This period includes the time required to prepare the necessary documents for the study, train study staff, set up the study sites, conduct study implementation and data collection and management procedures, clean and analyze data, and prepare the results for publication and presentation.

SAMPLE SIZE

This study is a pilot study with the primary goal of gathering efficacy information regarding ESVS for increasing infant WAZ at 30 days of age, and the sample size is therefore justified by the degree of precision with which we want to estimate the efficacy of this intervention after

accounting for loss to follow up.

With respect to the primary aim, determining the impact of ESVS on WAZ at 30 days, the anticipated sample size of 324 infants (n=144 LBW infants and n=180 infants with low weight on Day 4 under equal randomization) would provide 80% or greater power per weight stratum for treatment group comparison to detect a difference of 0.20 with respect to the outcome of WAZ at Day 30 for the entire study cohort after accounting for up to 20% loss to follow-up. This means per site we will have 72 LBW infants and 90 low weight on Day 4 infants. This sample size will also allow the estimation of the mean difference between WAZ at Day 30 and WAZ at birth with 0.17 and 0.15 standard errors (SEs) by treatment group, respectively, for the entire study cohort.

In the final analysis, if the effect of the intervention does not differ by region, data will be pooled between the regions and analyzed together for greater power. We emphasize that this is a pilot

study with the primary goal of collecting preliminary evidence regarding the ESVS. Mothers will be enrolled along with their infants. Since infants who are twins or other multiples will be excluded, one mother will be enrolled for each infant enrolled. Therefore, our total sample size for the randomized trial is anticipated to be 324 infants and 324 mothers, or 648 individual participants in total.

Of note, on the day of study enrollment (day of birth), it will not be possible to know whether an infant will weigh less than 2600 g on Day 4 and therefore be eligible for randomization on Day

4. For that reason, we estimate we will need to enroll a total of 1064 infants on the day of birth and follow them through at least Day 4 in order to achieve our anticipated sample size of 180 infants with low weight on Day 4 and 144 infants with low birth weight on the day of birth. On Day 4, infants weighing ≥2600 g or with weight loss ≥10% of birth weight will exit the study, and those weighing <2600 g will be randomized to either the intervention or the control group and will be followed for the study duration of 6 months. Neurodevelopment will be assessed only in a subset of 80 babies who will be followed until 1 year of age.

STUDY ACTIVITIES

# RECRUITMENT

Local clinicians will inform mothers about the study and refer those who are interested to the study staff. Once a prospective mother-infant pair is referred, trained study staff will review the birth record in the hospital to confirm eligibility for screening. Once screening eligibility is confirmed, trained study staff will initiate contact with mothers, verbally introduce screening for the study, assess potentially consenting mothers for the understanding of the aim of screening for the study and of the aim of the study, and confirm that eligibility for screening has been met. If the mother agrees to participate in screening for the study, participant rights for her infant and her will be reviewed, and consent will be obtained from the mother for the inclusion of her infant and herself in the screening. If the mother-infant pair completes screening and does not meet study entry criteria, no further study activities will occur. If the mother-infant pair completes

screening and does meet study entry criteria, trained study staff will describe the study to the mother in detail, assess her for the understanding of the aim of the study and confirm that eligibility for study participation has been met. If the mother agrees to participate in the study, participant rights for her infant and her will be reviewed, and consent will be obtained from the mother for the inclusion of her and her infant in the study.

# RANDOMIZATION

PRIMES will use a randomized controlled trial design. Randomization will be stratified by country (Guinea-Bissau or Uganda) and by weight at time of randomization (Day 0 weight 2000- 2249g, Day 0 weight 2250-2499g, Day 4 weight 2250-2424g, Day 4 weight 2425-2599g).

While study staff and mothers will not be blinded, randomization to study group will occur after enrollment and the study staff will have no foreknowledge of group assignment, so there will be complete allocation concealment. We target to enroll a total of 72 LBW infants per site and a total of 90 infants experiencing low weight on Day 4 per site. Under equal randomization, this will lead to n=36 and n=45 for each treatment group per country (Guinea-Bissau or Uganda) and per risk-group stratum (LBW or low weight on Day 4), respectively.

# CLINICAL MANAGEMENT OF STUDY PARTICIPANTS

With respect to clinical management of infant participants, all infants enrolled will receive usual care by their usual care providers. PRIMES will not provide clinical care and will refer infants with concerning signs or symptoms to the local health care provider or hospital for management. With respect to clinical management of mother participants, all mothers enrolled will receive usual care by their usual care providers. PRIMES will not provide clinical care and will not assess signs and symptoms in mothers. If study staff identify overtly concerning signs or symptoms in mothers, study staff will refer mothers to the local health care provider or hospital for management.

# STUDY VISITS

Dates for follow-up visits are calculated from Day 0, which is defined as the date of birth. Day 1 is defined by PRIMES as the calendar day after the birth has occurred (e.g. if an infant is born on Monday, then Day 1 is defined as Tuesday). Most infants will be enrolled on Day 0, but those born in close proximity to midnight may have their first study visit on Day 1. Study visits can occur at an agreed upon location such as the community health center or hospital or at the research center or the infant’s home. All visits will be documented in the appropriate study forms. See additional details of study visits in APPENDIX: SCHEDULE OF STUDY VISITS,

summarized as follows:

**Day 0:** Obtain informed consent for study participation within 24 hours of birth. Once informed consent for study participation obtained, assign study identification number, collect infant clinical and demographic information, weight, length, MUAC, head circumference, dietary survey, stool sample and collect mother’s contact information, weight, height and MUAC.

**Day 4:** Collect infant weight, length, MUAC, head circumference, dietary survey, and AEs and maternal and infant hemoglobin.

**Day 14:** Collect infant weight, length, MUAC, head circumference, dietary survey and AEs.

**Day 30:** Collect infant weight, length, MUAC, head circumference, dietary survey, stool sample and AEs.

**Day 60:** Collect infant weight, length, MUAC, head circumference, dietary survey and AEs.

**Day 90:** (**3 months):** For some infants, collect infant weight, length, MUAC, head circumference, dietary survey and AEs and assess with the Hyperfine portable MRI.

**Day 180 (6 months):** Collect infant weight, length, MUAC, head circumference, dietary survey, stool sample and AEs. For some infants, assess with the Bayley and Hyperfine portable MRI. Study exit for infants not receiving assessment with the Bayley and Hyperfine portable MRI.

**Day 365 (1 year):** Collect infant weight, length, MUAC, head circumference, dietary survey and AEs. Assess with Bayley, Hyperfine portable MRI and conventional (fixed) MRI. Study exit.

STUDY TERMINATION VISIT

The Day 180 (6 month) visit will serve as the study termination visit for infants not receiving neurodevelopmental assessment. The following activities are required during the exit visit on Day 180:

1. Collect infant weight, length, head circumference, MUAC, and skinfolds.
2. Collect infant stool sample.
3. Collect report on infant dietary intake including breastfeeding.
4. Collect data on AEs.
5. Maternal height, weight and MUAC

The Day 365 (1 year) visit will serve as the study termination visit for any infants remaining in the study at Day 365. The following activities are required during the exit visit on Day 365:

- 1. Collect infant weight, length, head circumference, MUAC, and skinfolds.
  2. Collect report on infant dietary intake including breastfeeding.
  3. Collect data on AEs.
  4. Assess with Bayley, Hyperfine Swoop MRI and conventional (fixed) MRI.

MISSED VISITS

Maximum effort will be made to ensure that Day 4 visits are completed on Day 4; Day 14 visits are completed on Day 13, 14 or 15; Day 30 visits are completed on Day 29, 30 or 31; Day 60 visits are completed on Day 54-66; and Day 180 (6 month) visits are completed on Day 166-194. Visits not completed within these time frames will be documented as “missed”. For “missed” Day 4 visits, trained study staff will attempt to collect the measurements through Day 10. For “missed” Day 14 visits, trained study staff will attempt to collect the measurements through Day

1. For “missed” Day 30 visits, trained study staff will attempt to collect the measurements through Day 45. For “missed” Day 60 visits, trained study staff will attempt to collect the measurements through Day 80. For “missed” Day 90 visits, trained study staff will attempt to collect the measurements through Day 110. For “missed” Day 180 (6 month) visits, trained study staff will attempt to collect the measurements through Day 224. For “missed” Day 365 (1 year) visits, trained study staff will attempt to collect the measurements through Day 395. If successfully collected, such measurements collected for “missed” visits will be associated with the date and time of collection and will be included in the data as “additional study visits.” Regardless of “missed visits” and regardless of “additional visits,” the trained study staff will attempt to complete each remaining study visit as initially scheduled (e.g. if the Day 14 visit is completed on Day 24, trained study staff will attempt to collect the Day 30 study measurements on Day 29, 30 or 31).

# STUDY PROCEDURES

PRIMES study staff will be required to receive Good Clinical Practice (GCP) training as well as study-specific training which depending on the staff’s role in the study may include training in administering questionnaires, obtaining infant and maternal weight, length/height, MUAC and hemoglobin measurements, and or obtaining infant intestinal microbiota and deuterium oxide excretion for a subset of infants. Study staff will strictly follow the guidelines described in this protocol and in the standard operating procedures (SOPs).

**Measured infant weight:** To ensure scale accuracy for infant weight (the primary outcome measure), trained study staff will use only the Seca 334 scale and will obtain two infant weights during each study visit. If these duplicate weights vary by 10g or more, an additional 2 readings will be made. If additional readings are made, each weight that varies 10g or less from another weight will be averaged to determine infant weight. Infants will be weighed naked without pacifiers/dummies. See Measuring infant weight SOP for additional details.

**Birth weight:** In order to be included in the study, an infant must have a birth weight obtained at

<6 hours of age by trained study staff using a Seca 334 infant scale that has been calibrated within the past 7 days. See Measuring infant weight SOP for additional details.

**Measured infant length and MUAC:** Infant length will be measured using the Seca 416 infant stadiometer and infant MUAC measured with MUAC tapes. Two lengths and two MUAC measures will be obtained at each visit during which infant length and MUAC is assessed. If duplicate measures vary by 0.5 cm or more or 0.2 cm or more for length or MUAC, respectively, an additional 2 readings will be made. If additional readings are made, each length and MUAC, respectively, that varies 0.5 or 0.2 cm or less from another length or MUAC will be averaged to determine length and MUAC.

**Measured infant skinfold thickness:** Infant skinfold thickness will be measured twice at each visit during which skinfold thickness is assessed using standard skinfold calipers. If duplicate measures vary by 0.2 cm or more, an additional 2 readings will be made. If additional readings are made, each thickness that varies 0.2 cm or less from another will be averaged to determine thickness.

**Maternal weight, height and MUAC:** Maternal weight, height, and MUAC will be measured twice at each visit during which they are assessed using standard scales, stadiometers and MUAC tapes. If duplicate measures vary by 0.5 kg, 0.5 cm or 2mm or more for weight, height and MUAC, respectively, an additional two readings of that measure will be assessed. If additional readings are made, each weight, height or MUAC, respectively, that varies 0.5 kg, 0.5 cm or 2mm or less from another will be averaged to determine maternal weight, height and MUAC.

**Hemoglobin:** Hemoglobin will be measured using the HemoCue (Hb 201+ System).

**Data collection instrument:** Information including but not limited to clinical and demographic characteristics, breastfeeding frequency, issues relating to breastfeeding, use of supplementary foods and fluids, frequency of breastfeeding, urination and stooling, antibiotic use, and AEs including occurrence of diarrhea or vomiting, WHO general danger signs, hospitalization or death will be captured in a series of simple questions. All infants will be monitored at each study visit for signs or symptoms of illness or any growth problems. If infant death is ascertained at the Day 4, Day 14 or Day 30 assessments, trained study staff will administer the WHO neonatal verbal autopsy instrument; if infant death is ascertained at the Day 60,Day 180 (6 month) or day 365 assessments, trained study staff will administer the WHO child verbal autopsy instrument.19

**Infant intestinal microbiota:** Stool samples will be collected from infants at enrollment and on Day 30 and Day 180 and will be stored in DNA/RNA Shield Fecal Collection Tube containers (Zymo Research, Irvine, CA) and will be transported to the University of California Davis for analysis.

**Maternal milk composition:** Immediately following a Day 30 breastfeeding, consenting mothers will hand express 10 mL milk from each breast (total of 20 mL milk). Milk will be frozen and transferred to UCSF for future analysis of caloric content, creamatocrit and microbiota. Mothers in Uganda will sign a separate specimen collection consent form at the time of enrollment to indicate willingness to participate in milk collection for future analysis. For mothers in Guinea- Bissau, willingness to participate in milk collection will be included in the main study consent form.

***Infant breast milk intake (Deuterium oxide-to-the-mother):*** *The deuterium oxide-to-the-mother method is a gold standard to measure breast milk intakes in infants.20-22 The method takes advantage of the flow of the deuterium oxide from the mothers through breast milk into the body water of the infants. Based on how fast the deuterium oxide disappears from the mother and how fast the deuterium oxide appears and disappears from the infant, the amount of breast milk consumed by the infant can be calculated. Since deuterium is a natural occurring isotope of hydrogen and is found naturally in the water, beverages and foods consumed every day, the method is considered safe. Deuterium is found naturally in body tissues as well. Many studies have used the same method to measure breast milk intake in infants in the United States and around the world with no reported AEs on the mothers and infants.*

*This measure will be assessed in a subsample of mothers (n=60, with 30 at each site.) To assess this measurement, mothers will be seated to consume a small volume of deuterium oxide diluted in a beverage. Deuterium will not be administered undiluted and mothers will remain seated accompanied by trained study staff for 10 minutes after deuterium consumption. Samples of maternal and infant urine will be collected for sequential days following administration and will be stored for analysis. To further ensure accuracy for this measurement, in-person training in the administration and measurement of deuterium oxide and collection and storage of urine samples will be conducted on site prior to initiation of administration of deuterium oxide. If in- person training is not permitted or feasible we will instead consider using an alternate measurement for breast milk intake described below. See Deuterium oxide-to-the-mother collection SOP for additional details.*

***Infant breast milk intake (before-and-after test weights):*** *If in-person training for the use of deuterium oxide is not feasible we will measure infant breast milk intake using before-and-after test weights for 30 consecutive infants at each site. We will weigh the baby before and after each feeding for 24 hours and will record the differences in weight before and after each feeding without any change in clothing, diapers or blankets between the two weights. See Before-and- after Test Weight SOP for additional details. If pandemic-related constraints or other constraints do not permit either deuterium oxide or before-and-after test weights, we will not assess milk intake in this study.*

**Neurodevelopment:** For a subset of 80 infants in Uganda, neurodevelopment will be assessed by Hyperfine MRI /Swoop™ Portable MRI at 3, 6 and 12 months; by conventional (fixed) MRI at 12 months; and with the Bayley Scales of Infant and Toddler Development at 6 and 12 months.

- - Magnetic Resonance Imaging (MRI) uses a magnetic field, radio waves, and a computer to produce detailed pictures of the body’s internal structures. Conventional (fixed) brain MRI will be obtained once at 1 year of age in a subset of 80 Ugandan infants, in a hospital (Nsambya Hospital).
  - Hyperfine Swoop™ Portable MRI is a magnetic resonance imaging scanner system that uses a low magnetic field (0.065 Tesla) and can be plugged into a standard electrical wall outlet at a clinic and controlled through a wireless tablet. Designed to be simple to use, a

radiologist will be trained by the manufacturer on system operation, device navigation and safety. Hyperfine MRI will be obtained at 3, 6 and 12 months at the research clinic.

- - Bayley Scales of Infant and Toddler Development is the most commonly used assessment tool of neurodevelopment in early childhood and has been modified for use during infancy in LMIC. We will administer the Bayley scale to all infants at 6 and 12 months of age to assess the impact of the intervention on infant neurodevelopment.

# CALENDAR OF INFANT ASSESSMENTS

**Day 0/1**: Within 24 hours of birth, trained study staff will obtain informed consent from the mother for the participation of her and her infant. Trained study staff will then assign a study identification number to the family and will collect the mother’s contact information, and infant birth date, time and place, weight, length, MUAC, head circumference, type of birth and sex and stool sample, and will survey the mother regarding dietary intake since birth.

**Day 4:** Collect infant weight, length, MUAC, head circumference, dietary survey and AEs.

**Day 14:** Collect infant weight, length, MUAC, head circumference, dietary survey and AEs.

**Day 30:** Collect infant weight, length, MUAC, head circumference, dietary survey, stool sample and AEs.

**Day 60:** Collect infant weight, length, MUAC, head circumference, dietary survey and AEs.

**Day 90 (3 months)**: For some infants, collect infant weight, length, MUAC, head circumference, dietary survey and AEs and assess with Hyperfine MRI.

**Day 180 (6 month):** Collect infant weight, length, MUAC, skinfolds, head circumference, dietary survey, stool sample and AEs. For some infants, assess with Bayley Scales and Hyperfine MRI. For infants not receiving assessment with Bayley Scales and Hyperfine MRI, study exit.

**Day 365 (12 months):** Collect infant weight, length, MUAC, head circumference,dietary survey, AEs and assess with Bayley Scales, Hyperfine MRI and conventional (fixed) MRI. Study exit.

*ADDITIONAL ASSESSMENT OF MILK INTAKE VOLUME FOR 30 CONSECUTIVE INFANTS ASSESSED AT 60 DAYS OF AGE AT EACH SITE*

***Breast milk intake will be measured for 30 consecutive (as possible during the pandemic) infants assessed at 60 days of age at each site.*** *If in person training and monitoring are permitted and feasible at both sites, we will conduct in-person training on the use of deuterium oxide at both sites a. If this in-person training is successfully conducted, we will measure infant breast milk intake at 60 days of age by administering deuterium oxide at 60 days after birth to mothers of 30 consecutive infants at each site on Day 60 and collecting subsequent samples of maternal and infant urine between Day 60 and Day 75.*

*If in-person training is not permitted, we will not administer deuterium oxide to any mothers and will instead measure breast milk intake using before-and-after test weights over a 24 hour period at 60 days of age for 30 consecutive infants at each site. See SOPs for additional details on procedures for deuterium oxide and for the before-and-after test weights.*

# CALENDAR OF MATERNAL ASSESSMENTS

**Day 0/1 (Day of Infant Enrollment):** Trained study staff will collect data on the mother, including maternal height, weight, MUAC, age and educational attainment as well as marital status, water source, wealth index and household toilet practices.

**Day 30:** Trained study staff will collect up to 20 mL of maternal breast milk collected by mother using hand expression immediately after a breastfeeding. See Collecting expressed maternal breast milk SOP for additional details.

**Day 180 (6 months):** Trained study staff will collect data on maternal height, weight and MUAC. Study exit.

# TREATMENT ASSIGNMENT TO INTERVENTION OR CONTROL

After informed consent is obtained, trained study staff will access a password-protected secure program on a study mobile device that will randomly allocate treatment assignment to either ESVS or control.

A major study consideration is that some newborns randomly assigned to the recommendation of EBF will, in fact, actually receive some form of supplementation. The premise of our study is that the carefully managed supplementation of ESVS may benefit growth when compared to a recommendation of EBF that might result in either EBF or in unstructured supplementation with liquids of low nutritional value. The purpose of this pilot is to compare ESVS with the current standard of care of recommending EBF, with the recommendation followed by some and not by others. Therefore, our primary analysis will be intention-to-treat, which will inform the primary public health policy question of whether recommending ESVS improves growth.

# DATA COLLECTION

Data will be collected electronically. Study data including survey responses will be entered directly into study databases during the study visits.

Study staff will be trained on how to collect and document study measurements to standardize procedures and data collection across study sites. Periodic site reviews will identify any problems necessitating additional quality control measures. Participating dyads will be identified by collecting their names, phone number(s) and any known addresses at which they might be residing over the course of the study. In order to identify infant residence, various additional techniques may be used at sites including drawing maps to depict the home location, shadowing the mother and infant back to their home following hospital departure to identify location, and use of pre-assigned residential block numbers. All the above personal identifiers will be used by trained study staff to follow participants over the course of the study. Personal identifiers will be stored securely on a password-protected, secure server in Uganda. In Guinea-Bissau personal identifiers will be stored securely on paper files. Site investigators will also assign each dyad an ID number and provide each dyad with a laminated card containing the dates of follow up visits as well as the family ID number and/or a bar code to facilitate identification during study follow up.

# TRAINING

Before the study starts, all study staff will receive GCP and study-specific training to review all study procedures, protocol, data collection tools, informed consent process, and reporting requirements. Training will be conducted by a member of the investigative team or their delegated representative.

# ADVERSE EVENTS

We will collect data on AEs at each outcome study visit. The plan to collect AE information at outcome study visits allows for equivalent data collection in intervention and control infants, as the study team anticipates that AEs (both severe and non-severe), will occur among enrolled infants receiving ESVS at similar rates as clinical events of similar severity occur in the control population. AEs common in the local study populations include, but are not limited to: infectious diseases, dehydration, malnutrition and sudden unexpected death in infancy.

Serious Adverse Events

Serious adverse events (SAEs) will be defined as AEs that:

- Result in death;
- Are life-threatening AEs;
- Require inpatient hospitalization;
- Result in persistent or significant disability/incapacity: or
- Are important AND, based upon appropriate medical judgment, may jeopardize the health of the participating infant or require medical or surgical intervention to prevent one of the outcomes listed above.

For all SAEs, the relationship between the SAE and ESVS will be evaluated. For SAEs occurring among infants after study treatment randomization, an internal medical officer will assign a determination of the relationship of the SAE to ESVS. The internal medical officer’s determination will be final for all SAEs except death. For any death among infants after study treatment randomization to ESVS, an independent medical officer will make the final determination of relationship to ESVS.

Serious Adverse Event Relationship to ESVS

The relationship of all SAEs to ESVS will be assessed as follows23:

Definitely related: SAE and administration of ESVS are related in time, and a direct association can be demonstrated with ESVS.

Probably related: SAE and administration of ESVS are reasonably related in time, and the SAE is more likely explained by ESVS than by other causes

Possibly related: SAE and administration of ESVS are reasonably related in time, and the SAE can be explained equally well by causes other than ESVS

Probably not related: a potential relationship between SAE and administration of ESVS could exist, but is unlikely, and the SAE is most likely explained by causes other than ESVS

Not related: SAE is clearly explained by another cause unrelated to administration of ESVS. For participants randomized to ESVS, reportable events must have documentation to support the determination of “not related.”

# SAFETY REPORTING

All SAEs must be reported by the site to the local PI or local co-PI within 24 hours of receiving information from the mother/representative. All SAEs must be entered into the PRIMES database within 72 hours of receiving the information from the mother/representative. Reporting requirements to the respective institutional review board (IRB) will be followed as determined by each IRB’s requirement. The UCSF team will report all SAEs to the UCSF IRB within 7 days.

# STUDY DISCONTINUATION

The trial may be discontinued at any time by the investigators, site regulatory authorities, or IRBs.

# DATA MANAGEMENT

Family ID will be assigned sequentially beginning at 0001 for each site. If a site is enrolling in more than one location of enrollment (hospital, clinic or birthing center), family ID will be assigned sequentially with the first digit varying by site (i.e. beginning at 0001 at the first site, 1001 at the second site, 2001 at the third site, etc.).

Data management will be centralized at UCSF. We will design standardized electronic data collection instruments that can be used at all PRIMES study sites so that collected data can be uploaded at least weekly in a de-identified and timely manner to a secure, password-protected server at UCSF. Once uploaded, data will be queried and cleaned at UCSF, with feedback provided to site study staff regarding any changes needed to optimize data utility. The study site investigators will be responsible for maintaining, and storing securely, complete, accurate and current study records throughout the study including signed informed consent documents. The study sites will maintain appropriate study records for the study in compliance with all locally applicable IRB, regulatory, sponsoring organization and institutional requirements for the protection of confidentiality of participants. Direct access to study data will be granted to authorized representatives from UCSF and the co-investigators, and the local and national IRB and regulatory authorities for monitoring and/or audit of the study to ensure compliance with protocol and high-quality data. Sites will retain all study records for at least three years after study closure. Study records will not be destroyed prior to receiving approval from UCSF. Applicable records include site enrollment documents, surveys, reports, and informed consent forms. Site investigators will work together with UCSF investigators to complete data validation and will have the opportunity to collaborate with UCSF investigators and the co-investigators for data analysis.

# STUDY MONITORING

Primary monitoring of overall study site conduct will be the responsibility of the local principal investigator (PI), who will also have primary responsibility for addressing any barriers to complete and valid data collection. Sites must alert the UCSF team if unexpected concerns arise including any concerns about integrity of data, loss of confidentiality or participant safety.

UCSF personnel in conjunction with co-investigators will monitor and audit the study in accordance with the currently approved protocol, relevant regulations, and SOPs. Sites will be regularly contacted *and visited (if feasible during COVID-19 pandemic)* by the study monitoring team for the purpose of training, study monitoring and refresher training if needed. *During such monitoring visits, site facilities may be inspected and study staffing, equipment, and study procedures reviewed. If pandemic-related conditions preclude in-person visits, live video monitoring using a secure platform will be used to inspect site facilities and confirm that study procedures including infant measurements are conducted in accordance with the protocol. In addition, for mothers providing informed consent for live video monitoring, live video monitoring of enrollment, treatment assignment and follow up will be used if in-person monitoring is not feasible.* Upon request, study investigators and/or investigators from UCSF may inspect the various records of the study. Participant confidentiality will be maintained.

# DATA STORAGE

All data will be stored securely. Informed consent forms will be stored in a locked file cabinet in a locked office at the local site. All other data will be stored electronically on a Research

Electronic Data Capture (REDCap) database maintained, encrypted and password-protected by UCSF.

Study staff at the local sites will be equipped with hotspots to access the study REDCap database on Android phones or tablets and will directly enter data into the database as it is collected. If cellular or WiFi access is not available at the location of data collection (e.g., if a study visit occurs at a participant’s home and the hotspot does not function), study staff will enter data into REDCap during the study visit and will then promptly return to a location with cellular or WiFi access (e.g., the research site) to upload the data to the database. Paper case report forms (CRF) will be available in the event the software malfunctions. If a paper CRF is used, study staff will enter data from the CRF into the database as soon as possible and within 72 hours and will send a scanned, de-identified copy of the CRF to UCSF within 7 days.

# STATISTICAL ANALYSIS

Our primary analysis will be intention-to-treat, which will inform the primary public health policy question of whether recommending ESVS improves growth. In order to account for our planned stratified randomization, we will use a permutation test (permuting the treatment assignment within the weight stratum) to determine the effect of ESVS on WAZ at 30 days of age, on WLZ at 30 days, on WAZ, WLZ and LAZ at 180 days, on white matter volume and total brain volume at 90, 180 and 365 days, on Bayley scale scores at 180 and at 365 days, on intestinal microbiota at 30 days, and on breastfeeding duration and receipt of non-breastmilk dietary intake between birth and 6 months.

For the outcome of intestinal microbiota, we will use cluster and eigenstructure analyses (e.g., principal component analysis) for descriptive analysis of 16S rRNA gene abundance data, followed by adonis permutation tests for significance of associations with continuous or factor variables or Mantel tests for associations between distance matrices. For example, treatment group is an important factor variable; a significant association of the 16S rRNA gene profile with group membership would suggest an effect of ESVS on microbiota. Specific hypotheses generated by consideration of our preliminary data will be tested either by non-parametric tests (e.g., Kruskal-Wallis test for different abundance of *B.infantis* in experimental groups) or by linear models (e.g., for testing the relationship between bacterial species). Appropriate correction for multiple comparisons will be employed, such as calculation of false discovery rates. Additionally, exploratory analyses will be undertaken with supervised machine learning approaches, in order to identify new and unexpected features unique to the intestinal environment of ESVS recipients. For example, random forests24 will be used to identify important features separating the microbiotas of ESVS recipients from controls.

For the outcomes of total white matter volume and total brain volume, we will use generalized linear regression to compare the ESVS group to the control group. For the outcomes of neurodevelopment as measured by the Bayley Scales of Infant and Toddler Development, we will compare Bayley score between the ESVS and control groups using a generalized linear mixed effects model that accounts for measurements missing due to loss to follow up under the standard missing at random assumption.

# DATA SAFETY AND MONITORING PLAN (DSMP)

Since both EBF and breastfeeding with supplementation are common in the newborn period, we anticipate that the risks of study participation will not exceed the ordinary risks of the newborn period. We therefore plan the following timetable for the entire study for assessment of any unanticipated risks associated with randomization:

After 81 dyads (25%) have completed primary outcome assessment at 30 days of age, the PRIMES data and safety monitoring board (DSMB) will review the collected data and compare the intervention and control groups with respect to serious adverse events (SAEs) and mortality. If the collected data demonstrate a statistically significant (p<0.005) relationship between treatment assignment and SAEs or between treatment assignment and mortality, the DSMB will recommend that the study should be stopped. If the collected data demonstrate a trend towards a relationship between treatment assignment and SAEs or between treatment assignment and mortality (0.005 ≤ p <0.01), the DSMB will recommend that the study’s informed consent should be revised to include additional information on possible adverse consequences (i.e., on SAEs or mortality) of the study.

After 162 dyads (50%) have completed primary outcome assessment at 30 days of age, the PRIMES DSMB will review the collected data and compare the intervention and control groups with respect to SAEs and mortality. If the collected data demonstrate a statistically significant (p<0.01) relationship between treatment assignment and SAEs or between treatment assignment and mortality, the DSMB will recommend that the study should be stopped. If the collected data demonstrate a trend towards a relationship between treatment assignment and SAEs or between treatment assignment and mortality (0.01 ≤ p <0.02), the DSMB will recommend that the informed consent should be revised to include additional information on possible adverse consequences (i.e., on SAEs or mortality) of the study.

In addition to the above, the PRIMES study team will conduct interval monitoring of mortality of randomized infants after every 20 new randomized dyads complete outcome assessment at 30 days of age. During the period prior to achieving the milestone of completion of primary outcome assessment for 162 dyads (50%), if interval mortality monitoring shows that the infant mortality differs between the groups with p<0.005 with a **two-tailed hypothesis test,** the team will stop enrollment and convene the DSMB. At that time, the DSMB may recommend continuation or early stopping of the trial. After achieving the milestone of completion of primary outcome assessment for 162 dyads (50%), if interval mortality monitoring shows that the infant mortality differs between the groups with p<0.01 with a **two-tailed hypothesis test,** the team will stop enrollment and convene the DSMB. At that time, the DSMB may recommend continuation or early stopping of the trial.

# DATA SAFETY AND MONITORING BOARD

Prior to initiation of enrollment, the investigators will assemble an independent, 5-person DSMB

that will consist of at least one member with expertise in pediatrics, at least one member with expertise in nutrition, at least one member with expertise in global health and at least one member with expertise in biostatistics. The DSMB will meet once prior to study initiation and then again after the first 81 dyads have completed primary outcome assessment at 30 days of age, and then for a final meeting after 162 dyads have completed primary outcome assessment at 30 days of age. At these meetings, the DSMB will review available data and, in accordance with the DSMB charter, make determinations regarding whether the study should stop, continue as planned or continue with a revised informed consent form.

# RESULTS PRESENTATION AND DISSEMINATION

The primary results of this research *will be presented by the UCSF PI at an appropriate academic conference* and through at least one published manuscript with a detailed description of the background, methods, results, and conclusion. The specific format and details of the primary manuscript will be in accordance with the requirements of the publishing journal.

Secondary results may be presented and written up by coinvestigators.

The investigators will be involved in reviewing drafts of the manuscripts, abstracts, press releases and any other publications arising from the study. Authors will acknowledge that the study was funded by the BMGF. Authorship will be determined in accordance with UCSF and journal guidelines and other contributors will be acknowledged.

Dissemination will occur via peer-reviewed scientific literature *and at international conferences*.

# ETHICAL CONSIDERATION AND CONSENT

This study will be conducted in compliance with the protocol and all applicable IRB reviews. All paper records, including signed informed consent, will be kept in a locked office at the study site. No personal identifiers will be transferred to UCSF. The de-identified measurements will be stored securely in electronic form for a minimum of 10 years after the study.

INSTITUTIONAL REVIEW BOARDS (IRBs) AND INDEPENDENT ETHICS COMMITTEES (IECs).

The PI will obtain approval from the UCSF IRB for the hypotheses, objectives, study design, study staff and sites and will request modifications to the approval as needed. Local PIs will submit the protocol and obtain approval from all necessary ethical and regulatory bodies including their local IRB and all applicable regulating authorities. Approval will be sought from the Guinea-Bissau National Committee on Ethics in Health Comite Nacional de Etica na Saude, the Higher Degrees, Research and Ethics Committee of Makerere University, and the Uganda National Council of Science and Technology.

INFORMED CONSENT

Informed consent is the process of ensuring that mother participants fully understand what will and may occur during participation of the infant and the mother in the study. In obtaining and documenting informed consent, the site investigators will comply with applicable local and domestic, ethical and regulatory requirements and will inform the participant that the study involves research, describe the study’s risks, benefits and alternatives and the confidentiality of records, provide contact information for answers to pertinent research questions and state the voluntary nature of participation.

After completing the above procedures, trained study staff will assess for participant understanding using open-ended questions to elicit any areas needing additional clarification and will provide additional clarification to the participant if such areas are identified. Potential study participants will be encouraged to ask questions and to exchange information freely with trained study staff. Potential participants will be informed that there will be no repercussions for not participating. If after all of the above, the potential participant indicates willingness to enroll, informed consent will be obtained from the study participants by signature or thumbprint of the consent form. The consent forms will include the purpose of the study, a description of the procedures to be followed and the risks and benefits of participation and will give individuals all the relevant information they need in a language they understand to decide whether to participate, or to continue participation, in this study. Before any participant begins participation in the study, it is the site investigators’ responsibility to ensure that informed consent has been obtained.

All consent materials will be approved by the appropriate IRB prior to use. The informed consent will provide information about the purpose of the study and what participation implies and participants can decide whether or not they want to participate or continue to participate in the study. Participants will be given a copy of the consent forms. Mothers will provide consent for themselves and their infant.

# RISKS TO PARTICIPANTS

Overall risks

This is a randomized trial that is investigating the efficacy of ESVS. It is possible that ESVS might increase the risk of diarrhea, infectious disease and breastfeeding cessation, exposing infants in the ESVS intervention group to increased risk of AEs, hospitalization or death compared to infants in the EBF control group. It is also possible that ESVS promotes healthygrowth and that infants in the EBF control group could experience higher rates of growth failure, with an increased risk of AEs, hospitalization or death.

Diarrhea, infectious diseases, breastfeeding cessation, and growth failure are common risks in the newborn period in LMIC, and both breastfeeding with supplementation and EBF are common. For this reason, the risks to study infant participants are not anticipated to differ from the ordinary risks of the newborn period. The purpose of PRIMES is to determine if the incidence of any of these risks differs between the intervention and control groups, with the hypothesis being that these metrics are improved in the intervention group compared to the control group.

In addition, given the current COVID-19 pandemic, risks to participants could potentially include transmission of COVID-19 between study staff and participants. The mitigation plan for this risk is described below in the section “Protection Against Risks.”

Claustrophobia is a risk of both conventional (fixed) MRI and portable, point-of-care MRI. Conventional (fixed) MRI also has projectile risk from the strong magnetic fields used, and acoustic risk from the noise generated. This study will not use sedation for MRI so the risks associated with sedation are not risks of this study.

PROTECTION AGAINST RISKS

Protection against overall risk: In order to minimize the overall risk of AEs, SAEs, and death, eligibility criteria for this study have been carefully selected and a robust safety monitoring scheme is in place. The infant with growth deficiencies most at risk of adverse consequences from ESVS will be excluded from this study, including those with birth weight <2000g, those with weight loss ≥10% on Day 4, those with congenital abnormalities and those with WHO danger signs. Safety monitoring for this study includes frequent outcome assessment and facilitated referral to medical care for those with concerning signs or symptoms or measured weight loss ≥10% of birth weight. When these are identified, study staff in Guinea-Bissau and Uganda, respectively, will contact Dr. Carlito Bale at Simon Mendes Hospital and Dr. Victoria Nankabirwa at Makerere University, respectively; Dr. Bale and Dr. Nankabirwa will provide medical consultation and facilitated referral as appropriate. All SAEs will be assisted to receive prompt clinical care, as appropriate.

All infants will receive the standard-of-care recommendation to breastfeed based on UNICEF recommendations. Because study participants will receive extra instruction in UNICEF recommendations as well as additional weight monitoring, study infants are anticipated to be better nourished than infants who do not participate, irrespective of random treatment assignment.

Coercion: Caregivers may feel coerced to enroll in the study in order to receive care for their infant within a research setting, which may be perceived as of a higher quality than the standard of care. In order to minimize the risk of coercion, study staff will not be recruiting participants directly. Instead, local clinicians will inform mothers about the study and refer only those who

are interested to the study staff. During the informed consent process, study staff will emphasize that infants will have access to standard medical care whether enrolled in the study or not.

Specimen collection: The study involves blood specimen sampling by heelstick (infant) or fingerprick (mother). These can cause pain and bruising at or around the blood draw site. In order to minimize the risks associated with heelstick (infants) or fingerstick (mothers), all study

staff who will be collecting specimens from infants in the study will be trained in the appropriate procedures and supervised accordingly.

Loss of privacy and/or discomfort or embarrassment: Participants in the study will be queried regarding infant feeding practices, HIV status, marital status, and indices of wealth. Responding to these queries may result in a loss of privacy, discomfort or embarrassment for participants. To protect against these risks, interviews and exams will be conducted in private when feasible. If private interview and/or exam is not feasible, study staff will ask the mother if she would prefer to skip any portion of the study visit or defer data collection until privacy is feasible. All data will be stored securely, either in a locked file cabinet in a locked office or using password- protection for digital media. All participants will be reminded that they can discontinue study participation at any time, and we will use staff trained in the appropriate procedures.

Risk of transmission of COVID-19: Since study participants will be measured in person by study staff, transmission of COVID-19 is a risk of this study. To mitigate this risk, all local and national COVID-19 infection control guidelines and precautions will be followed. The study team will take all precautions recommended by national and local authorities during the study visit, which will include but not be limited to: study staff will not work if they have any signs or symptoms of illness, including fever, cough, rhinorrhea, loss of smell or taste, or any other concern identified as related to COVID at the time (assessed daily and documented); study staff will wear masks and gloves during all participant interactions; study staff will clean hands with soap and water or alcohol-based hand sanitizer just prior to physical contact with participants; and study visits will take place in the open air (outdoors) when feasible.

Risk of MRI: Using the FDA-approved Hyperfine Swoop portable, point-of-care MRI greatly reduces the risks of conventional (fixed) MRI. First, it uses only a tenth of the magnetic field of conventional MRI removing projectile risk and allowing parents to accompany children.

Like conventional (fixed) MRI, it does not use radiation so there is no risk of radiation exposure. Second, it operates with a maximum noise level of 60 decibels, similar to normal conversation, so there is no acoustic risk. Third, the body of the machine is made of a screen material so that infants can easily see the room and their families, reducing the risk of claustrophobia. Parents can touch infants during point-of-care MRI if needed. To minimize risks during both conventional MRI and portable point-of-care MRI, sedation will be not be used for assessments. For conventional MRI, to reduce projectile risk, all assessments will be conducted at Nsambya Hospital by experienced, trained clinical personnel following standard safety procedures; to reduce acoustic risk from conventional MRI, all children will be provided with acoustic protection.

#

# BENEFITS TO PARTICIPANTS

Some study infants may benefit from close monitoring of weight, allowing prompt referral of growth problems. Infants randomly assigned to ESVS might benefit from the additional nutrition provided by ESVS. Infants randomly assigned to control might benefit from the EBF recommendation. All participants might potentially benefit from a feeling that they have contributed to improved understanding of breastfeeding practices and infant weight patterns in LMIC.

# PARTICIPANT CONFIDENTIALITY

The site investigators will ensure that each participant’s confidentiality is maintained. Personal identifiers will not be included in study reports. Study procedures will be conducted to protect participant’s privacy and confidentiality to the extent possible. All data will be de-identified prior to publication or dissemination

# REIMBURSEMENT

It is anticipated that some study visits will occur at home and others will occur at the hospital or at the study site. Participants will receive reimbursement for the cost of any study-related travel to the hospital or study site. Additionally, all randomized study infant-mother participants will receive diapers for stool collection and transportation reimbursement appropriate for the location chosen.

# POSSIBLE CONSTRAINTS

An anticipated study challenge will be ensuring quality and consistency of implementation. We plan to provide training, supervision, and oversight to ensure quality and harmonized study procedures across the sites. Another potential challenge includes following up with dyad participants. Recognizing that some infants may not complete the follow-up study visits, we plan to have trained study staff attempt to locate participants who miss their follow-up appointments and offer the option of follow-up at either the study site or the participant home to maximize participant convenience. Another potential challenge may be the COVID-19 pandemic, which might disrupt study activities in various ways including but not limited to interrupting enrollment, interfering with planned follow-up procedures and delaying or precluding in-person UCSF and co-investigator monitoring visits. To address the possibility of COVID-19 pandemic- related constraints on study activities, we have included additional pandemic-related protections against risks for study staff and participants and have identified components of the protocol that will be optional if COVID-19 pandemic-related constraints do not permit, including assessment of milk intake and in-person training and monitoring. These optional components of the study protocol are designated throughout in italics.

# WITHDRAWAL AND EARLY TERMINATION

Participants will not be required to comply with the randomly assigned treatment intervention; any participant can withdraw from the study for any reason at any time.

Infant’s caregivers may voluntarily withdraw themselves and their infants from the study for any reason at any time, including COVID-19 pandemic-related concerns. Any participant withdrawals or early termination will be documented in the appropriate study forms. Early termination or withdrawal from the study will not affect any care the participant may be receiving from the hospital or birth facility.

# PARTICIPANT RETENTION

To improve retention, site investigators will fully explain the purpose of the study and its importance for improving local health to local leaders prior to beginning enrollment and to parents and healthcare workers during the recruitment of infant-mother participants; site investigators might also team up with local healthcare workers to maintain contact with participants and remind them of study schedules.

# BIOHAZARD CONTAINMENT

Appropriate secretion precautions will be employed by all trained study staff. Biohazardous waste will be contained and discarded appropriately following all applicable local, regional and national regulations.

The use of safety equipment combined with good procedures and practices will help reduce the risks involved in dealing with biosafety hazards. Standard precautions will always be followed; barrier protection (gloves) should be used whenever samples are obtained from participants and when processing specimens. Adequate and conveniently located biohazard containers should be available for disposal of contaminated materials.

# PERSONNEL

Study staff will vary depending on the sites and will include GCP certified individuals with study-specific training capable of carrying out the above study activities. Each site’s study staff will include at minimum one study physician and one individual who can translate information

into English from any local languages used to collect data.

# SUPPLIES

Supplies for this study include the following:

Seca 334 infant scale only (no other infant scale should be used) 1, 2 and 5 kg Calibration Weights

MUAC tapes- infant and adult Seca 416 stadiometer

Adult scale and stadiometer

Android phones for REDCap data collection Tablets/laptops for communication

Standard office supplies including binders, paper, pens Hemoglobinometers and supplies

Fecal collection tubes (Zymo Research DNA/RNA Shield Fecal Collection Tube containers) Milk collection tubes

Masks Gloves

# BUDGET

Funds will be disbursed by UCSF.

# REFERENCES

1. UNICEF/WHO/World Bank Group. Joint Child Malnutrition Estimates. 2018; <http://www.who.int/nutgrowthdb/2018-jme-brochure.pdf?ua=1>.
2. Smith LC, Haddad L. Reducing Child Undernutrition: Past Drivers and Priorities for the Post-MDG Era. *World Development.* 2015;68:180-204.
3. UNICEF/WHO. Baby-Friendly Hospital Initiative: Revised, Updated and Expanded for Integrated Care, Section 1, Background and Implementation, Preliminary version. 2006.
4. World Health Organization. Nutrition: Exclusive breastfeeding. 2018; <http://www.who.int/nutrition/topics/exclusive_breastfeeding/en/>. Accessed May 19, 2018.
5. Breastfeeding and the use of human milk. *Pediatrics.* 2012;129(3):e827-841.
6. Mwangome M, Ngari M, Bwahere P, et al. Anthropometry at birth and at age of routine vaccination to predict mortality in the first year of life: A birth cohort study in BukinaFaso. *PLoS One.* 2019;14(3):e0213523.
7. Bwenge Malembaka E, Tumwine JK, Ndeezi G, et al. Effects of complementary feeding on attained height among lower primary school-aged children in Eastern Uganda: A nested prospective cohort study. *PLoS One.* 2019;14(2):e0211411.
8. de Onis M, Branca F. Childhood stunting: a global perspective. *Matern Child Nutr.*

2016;12 Suppl 1:12-26.

1. Miller AC, Murray MB, Thomson DR, Arbour MC. How consistent are associations between stunting and child development? Evidence from a meta-analysis of associations between stunting and multidimensional child development in fifteen low- and middle-income countries. *Public Health Nutr.* 2016;19(8):1339-1347.
2. Black RE, Victora CG, Walker SP, et al. Maternal and child undernutrition and overweight in low-income and middle-income countries. *Lancet.* 2013;382(9890):427-451.
3. Walson JL, Berkley JA. The impact of malnutrition on childhood infections. *Curr Opin Infect Dis.* 2018;31(3):231-236.
4. Ajayi OR, Matthews GB, Taylor M, et al. Structural Equation Modeling of the Effects of Family, Preschool, and Stunting on the Cognitive Development of School Children. *Front Nutr.* 2017;4:17.
5. Fadnes LT, Nankabirwa V, Engebretsen IM, et al. Effects of an exclusive breastfeeding intervention for six months on growth patterns of 4-5 year old children in Uganda: the cluster- randomised PROMISE EBF trial. *BMC Public Health.* 2016;16:555.
6. Feldman-Winter L, Kellams A, Peter-Wohl S, et al. Evidence-Based Updates on the First Week of Exclusive Breastfeeding Among Infants >/=35 Weeks. *Pediatrics.* 2020.
7. Flaherman VJ, Aby J, Burgos AE, Lee KA, Cabana MD, Newman TB. Effect of early limited formula on duration and exclusivity of breastfeeding in at-risk infants: an RCT. *Pediatrics.* 2013;131(6):1059-1065.
8. Flaherman VJ, Narayan NR, Hartigan-O'Connor D, Cabana MD, McCulloch CE, Paul IM. The Effect of Early Limited Formula on Breastfeeding, Readmission, and Intestinal Microbiota: A Randomized Clinical Trial. *J Pediatr.* 2018;196:84-90 e81.
9. Quigley MA, Carson C, Sacker A, Kelly Y. Exclusive breastfeeding duration and infant infection. *Eur J Clin Nutr.* 2016;70(12):1420-1427.
10. Duijts L, Jaddoe VW, Hofman A, Moll HA. Prolonged and exclusive breastfeeding reduces the risk of infectious diseases in infancy. *Pediatrics.* 2010;126(1):e18-25.
11. World Health Organization. Manual for the training of interviewers on the use of the 2016 WHO VA instrument. Geneva2017.
12. Butte NF, Wong WW, Patterson BW, Garza C, Klein PD. Human-milk intake measured by administration of deuterium oxide to the mother: a comparison with the test-weighing technique. *Am J Clin Nutr.* 1988;47(5):815-821.
13. Bandara T, Hettiarachchi M, Liyanage C, Amarasena S, Wong WW. The Deuterium Oxide-to-the-Mother Method Documents Adequate Breast-Milk Intake among Sri Lankan Infants. *J Nutr.* 2015;145(6):1325-1329.
14. Galpin L, Thakwalakwa C, Phuka J, et al. Breast milk intake is not reduced more by the introduction of energy dense complementary food than by typical infant porridge. *J Nutr.* 2007;137(7):1828-1833.
15. U.S. Department of Health and Human Services NIoH, National Institute of Allergy and Infectious Diseases, Division of AIDS,. *Table for Grading the Severity of Adult and Pediatric Adverse Events, Corrected Version 2.1.* 2017.
16. Breiman L. Random forests. *Machine learning.* 2001;45(1):5-32.

# APPENDIX

APPENDIX: SCHEDULE OF STUDY VISITS

|  | Day of enrollment | Day 4 | Day 14 | Day 30 | Day 60 | *Day 90 (in a subsample of 80 participants in Uganda)* | *Days 60-75 (In a subsample of 30 participants at each site)* | Day 180  (6  months) | Day 365 (in a subsample of 80 participants in Uganda) |
| --- | --- | --- | --- | --- | --- | --- | --- | --- | --- |
| Eligibility assessment | X |  |  |  |  |  |  |  |  |
| Family ID # | X |  |  |  |  |  |  |  |  |
| Participant contact information | X |  |  |  |  |  |  |  |  |
| Informed consent | X |  |  |  |  |  |  |  |  |
| Infant DOB | X |  |  |  |  |  |  |  |  |
| Infant TOB | X |  |  |  |  |  |  |  |  |
| Infant place of birth | X |  |  |  |  |  |  |  |  |
| Delivery method | X |  |  |  |  |  |  |  |  |
| Infant sex | X |  |  |  |  |  |  |  |  |
| Infant weight | X | X | X | X | X | X |  | X | X |
| Infant length | X |  | X | X | X | X |  | X | X |
| Infant hemoglobin |  | X |  |  |  |  |  |  |  |
| Infant dietary survey |  | X | X | X | X | X |  | X | X |
| Adverse events |  | X | X | X | X | X | X | X | X |
| Stool sample | X |  |  | X |  |  |  | X |  |
| Maternal and infant urine |  |  |  |  |  |  | X |  |  |

| samples for deuterium oxide analysis |  |  |  |  |  |  |  |  |  |
| --- | --- | --- | --- | --- | --- | --- | --- | --- | --- |
| *Maternal milk samples* |  |  |  | *X* |  |  |  |  |  |
| Maternal hemoglobin |  | X |  |  |  |  |  |  |  |
| Maternal height | X |  |  |  |  |  |  | X |  |
| Maternal weight | X |  |  |  |  |  |  | X |  |
| Maternal age | X |  |  |  |  |  |  |  |  |
| Mother’s educational attainment | X |  |  |  |  |  |  |  |  |
| Maternal marital status | X |  |  |  |  |  |  |  |  |
| Water source | X |  |  |  |  |  |  |  |  |
| Household toilet | X |  |  |  |  |  |  |  |  |
| Head circumference | X | X | X | X | X | X |  | X | X |
| Skinfolds |  |  |  |  |  |  |  | X | X |
| Infant MUAC | X |  | X | X | X | X |  | X | X |
| Maternal MUAC | X |  |  |  |  |  |  | X |  |
| Wealth index (Equity Tool) | X |  |  |  |  |  |  |  |  |
| Bayley Scales |  |  |  |  |  |  |  | X |  |
| Hyperfine MRI |  |  |  |  |  | X |  | X |  |
| Conventional MRI |  |  |  |  |  | X |  | X | X |

*As detailed above in the sections Study Procedures and Calendar of Assessments, if pandemic-related or other concerns make the measurement of milk intake infeasible, this component of the protocol will be eliminated

1. **Uganda screening consent form**

# University of California, San Francisco, in collaboration with:

**MAKERERE UNIVERSITY COLLEGE OF HEALTH SCIENCES SCHOOL OF MEDICINE RESEARCH AND ETHICS COMMITTEE (SOM-REC)**

**INFORMED CONSENT TO PARTICIPATE IN RESEARCH**

**Permission to Screen for Eligibility for the research study Preventing Infant Malnutrition (PRiM)**

**CO-INVESTIGATORS**

Victoria Nankabirwa, MBBS, PhD; Lecturer, Makerere University; +256755757460

Jolly Nankunda, MBChB, MMed Paediatrics, Senior Consultant Paediatrician, Mulago Women and Neonatal Specialized Hospital

Olive Jackie Namugga, MBBS, PhD (c); Research Fellow, Makerere University; [olivedejackie@yahoo.com](mailto:olivedejackie@yahoo.com)

Valerie Flaherman, MD, MPH ; Associate Professor of Pediatrics and Epidemiology and Biostatistics ; [valerie.flaherman@ucsf.edu](mailto:valerie.flaherman@ucsf.edu)

Susan Roberts, PhD ; Consultant to this project, and Professor of Nutrition, Tufts University; [susan.roberts@tufts.edu](mailto:susan.roberts@tufts.edu)

Amy Sarah Ginsburg, MD, MPH; IMG Consulting LLC; [messageforamy@gmail.com](mailto:messageforamy@gmail.com)

# Background and rationale/purpose for the study:

You and your infant are invited to be screened for participation in a research study led by the University of California, San Francisco in collaboration with Makerere University. This research study is about breastfeeding, infant growth, and nutrition supplementation. If you and your infant are screened and found to be eligible for participation, you will be asked to participate in this research study to see if giving small amounts of infant formula in addition to breastfeeding improves infant growth. Once you have all your questions answered about the screening, and if you agree to be screened, you will be asked to sign this form. If the screening identifies that you are eligible for participation, once you have all your questions answered about the study, and if you agree to join the study, you will be asked to sign another form to indicate your permission to participate in this study.

# Sponsors of the research project and the organizational affiliation of the researchers:

This study is sponsored by the Bill and Melinda Gates Foundation. Researchers in this study are affiliated with Makerere University in Uganda, and the University of California, San Francisco, USA.

# The estimated duration the research participant will participate in screening for the study:

The screening procedure will occur today and will take about 10 minutes.

# Procedures:

All study screening procedures will take place at the health center. If you choose to participate, your infant will be weighed today, and you will be interviewed about your ability to participate in the study and your health. If your infant weighs between 2000 g and 2885 g, and if you and your infant meet other health and eligibility criteria, you will be asked if you are willing to participate in the research study.

# Who will participate in screening for the study:

Mothers and newborns <6 hours old who are healthy and eligible to participate in the research study.

# Possible risks, discomforts, and inconveniences:

No medical risks are anticipated from participation in screening. There may be inconvenience in undressing the infant, and the screening procedure may take about 10 minutes of your time.

# Potential benefits:

There is no expected benefit to you or your infant from the screening procedure. There may be a public health benefit to understanding infant health in Uganda.

# Confidentiality:

Personal information will be collected about you and your infant, and it will be kept strictly in confidence. If screening shows you and your infant are eligible for study participation, you will be asked to participate in the study and more information will be given to you about confidentiality. If screening shows you are not eligible for study participation, or if you decline

to participate further after screening, all information collected about you will be destroyed today and will not be shared or used in any way.

# Alternatives:

Screening for this study is voluntary; you do not have to be screened for the study if you do not want to. Refusal to be screened for the study will not place you or your infant at risk of losing access to medical care. If you choose not to be screened for the study or if you decide to leave the study, you will continue to receive the exact treatment offered by the responsible authorities.

# Cost:

There will be no cost to you and/or your infant for the screening or study participation.

# Compensation for participation in the study/ Reimbursement:

There is no reimbursement for the screening.

# Questions about the study:

If you ever have questions about this study, need any more information, or if you/your infant have a health problem or are hurt in the study, you should contact study Principal Investigator, Dr. Victoria Nankabirwa at telephone number 0755757460. If you have any questions, you have a right to ask Dr. Nankabirwa or PRIMES study staff. Your questions should be answered clearly and to your satisfaction. You should not feel that you have to participate. If you decide not to participate, tell PRIMES study staff.

# Questions about participants’ rights/Contact information:

If you have questions about your rights as a research volunteer, you may contact the Chair of the Higher Degrees, Research and Ethics Committee (HDREC) (Dr: Suzanne Kiwanuka on

+256772886377). The study staff will be happy to help you contact the right person to answer any questions you have. If you have any questions, you may ask them now or later, even after screening for the study has started. If you wish to ask questions later, you may contact your local Principal Investigator Dr.Victoria Nankabirwa.

# Statement of voluntariness:

You and your infant’s participation in screening for this study is entirely voluntary. If you decide to have you and your infant participate, you will be asked to sign this consent form and you will receive a copy with important information such as names and phone numbers of the study team.

You do not have to participate in screening for this research study. It is your choice. You may agree to participate now and change your mind later and stop at any time for any reason. If you choose to stop, this will not affect your relationship with any of the researchers, your healthcare providers, or the Ministry of Health. If you do not wish to participate, you will continue to receive regular healthcare for you and your infant in the same way you did before. If you want to be removed or are removed from screening the study, any information collected from you before you were removed from screening for the study will not be used. If you wish to participate in screening for this study, you must sign this form.

# Ethical approval:

This screening has been approved by Makerere University School of Medicine Research and Ethics Committee, the Uganda National Council for Science and Technology and the University of California, San Francisco Institutional Review Board.

# Consent:

Your signature or thumbprint below means that you understand the information given to you about the study and in this consent form. If you sign or place your thumbprint on this form, it means that you agree that you and your infant can be screened to take part in the study. You are not giving up any of your and your child’s legal rights by signing this informed consent document.

**STATEMENT OF CONSENT/ASSENT**

........................................................................... has described to me what is going to be done, the risks, the benefits involved and my rights regarding this study. I understand that my decision to participate in this study will not alter my usual medical care. In the use of this information, my identity will be concealed. I am aware that I may withdraw at any time. I understand that by signing this form, I do not waive any of my legal rights but merely indicate that I have been informed about the research study in which I am voluntarily agreeing to participate. A copy of this form will be provided to me.

Name ………………………Signature/thumb print of participant Date ……

Name ………………………Signature of parent/guardian for minors (If applicable)…………

Date ……......

Name Signature of interviewer/Person obtaining informed consent

……………………Date ………………….

# Declaration of Impartial witness

**Only required if child’s mother or legally appointed representative is illiterate or if required by another party**

- I have read the entire Informed Consent Form.
- I was present when the study team member obtaining consent read and explained the information in this document to the child’s mother.
- The information provided to the child’s mother is accurate and complete.
- That the child’s mother was encouraged to ask questions.
- I am satisfied that the child’s mother fully understands the content of this informed consent document and has had all her questions satisfactorily answered.

Name………………………Signature of witness (if applicable)……………Date…………….

1. **Uganda enrolment consent form**

# University of California, San Francisco, in collaboration with:

**MAKERERE UNIVERSITY COLLEGE OF HEALTH SCIENCES SCHOOL OF MEDICINE RESEARCH AND ETHICS COMMITTEE (SOM-REC)**

**INFORMED CONSENT TO PARTICIPATE IN RESEARCH**

**Study participation for mothers and infants Title of the proposed study: Preventing Infant Malnutrition (PRIM)**

**CO-INVESTIGATORS**

Victoria Nankabirwa, MBBS, PhD; Lecturer, Makerere University; +256755757460

Jolly Nankunda, MBChB, MMed Paediatrics, Senior Consultant Paediatrician, Mulago Women and Neonatal Specialized Hospital

Olive Jackie Namugga, MBBS, PhD (c); Research Fellow, Makerere University; [olivedejackie@yahoo.com](mailto:olivedejackie@yahoo.com)

Valerie Flaherman, MD, MPH ; Associate Professor of Pediatrics and Epidemiology and Biostatistics; [valerie.flaherman@ucsf.edu](mailto:valerie.flaherman@ucsf.edu)

Susan Roberts, PhD ; Consultant to this project, and Professor of Nutrition, Tufts University; [susan.roberts@tufts.edu](mailto:susan.roberts@tufts.edu)

Amy Sarah Ginsburg, MD, MPH; IMG Consulting LLC; [messageforamy@gmail.com](mailto:messageforamy@gmail.com)

**Introduction to research studies:** A research study is designed to answer specific questions, sometimes about effectiveness of a medication or supplement. Being in a research study is different from being a patient. When you and your baby are patients, your personal doctor has a great deal of freedom in making decisions about your health care and your baby’s health care. When you and your baby are research subjects, the research staff will follow the rules of the research study (protocol) as closely as possible, without compromising your health or your baby’s health.

# Background and rationale/purpose for the study:

You and your baby are invited to participate in a research study led by the University of California, San Francisco in collaboration with Makerere University. This research study is about breastfeeding, infant growth, and nutrition supplementation. You and your baby are being asked to participate in this research study to see if giving small amounts of infant formula in addition to breastfeeding improves infant growth. Once you have all your questions answered about the study, and if you agree to join the study, you will be asked to sign this form.

# Sponsors of the research project and the organizational affiliation of the researchers:

This study is sponsored by the Bill and Melinda Gates Foundation. Researchers in this study are affiliated with Makerere University in Uganda, and the University of California, San Francisco, USA.

# The estimated duration the you and your baby will take to participate in the research project:

We ask you to participate in this study until your baby is 365 days old (1 year old). We anticipate that the total time commitment for this study will be approximately 3-7 hours depending on whether milk intake or brain development assessments are completed.

# Procedures:

All study procedures will take place either at the health center or at your home. If you choose to participate, you and your baby will be measured today, and you will be interviewed about your health and wellbeing. If your baby weighs less than 2.5 kg, the study team will assign you and your baby by chance either to breastfeeding with formula supplementation for the first 30 days after birth, or to standard of care feeding as stipulated by the Ministry of Health which is exclusive breastfeeding with no additional foods or fluids for 6 months.

If your baby weighs 2.5 kg or more today, a study nurse will meet you at your home or at the health center to weigh your baby in four days. If the study nurse weighs your baby in four days and finds that your baby weighs 2.6 kg or more, you and your baby will finish your study participation at that time. If the study nurse weighs your baby in four days and finds that your baby has lost 10% or more of its birth weight, you and your baby will finish your study participation at that time, and the study team will advise you how to care for your baby’s health. If the study nurse weighs your baby in four days and finds that your baby weighs <2.6 kg and has not lost 10% or more of its birth weight, the study team will assign you and your baby by chance either to breastfeeding with formula supplementation until your baby is 30 days old, or to standard of care feeding as stipulated by the Ministry of Health which is exclusive breastfeeding with no additional foods or fluids for 6 months.

Assignment to either group option occurs by chance, similar to flipping a coin. There is a 50% chance that you and your baby will be assigned to breastfeed with formula supplementation until your baby is 30 days old, and a 50% chance that you and your baby will be assigned to the standard of care feeding as stipulated by the Ministry of Health which is exclusive breastfeeding with no additional foods or fluids for 6 month .

If you and your baby are assigned to breastfeed with formula supplementation, the study staff will encourage you to breastfeed your baby frequently, and supplement with formula provided by the study following each breastfeeding until your baby is 30 days old. If you and your baby are assigned to the standard of care as stipulated by the Ministry of Health, the study staff will encourage you to breastfeed exclusively with no additional foods or fluids for 6 months. It is important that you consistently follow the requirements for your assigned study group in terms of breastfeeding and using formula supplementation.

Other study procedures will include:

Your weight and height will be collected today and in 180 days (6 months)

Your baby’s weight, height and head circumference will be collected today and in 4, 14, 30, 60 and 180 days from today and for some infants at 365 days from today.

You and your baby’s mid-upper arm circumference (MUAC)—For this procedure, a tape measure will be used to measure around you and your baby’s upper arm. Your MUAC will be collected today and at 180 days (6 months). Your baby’s MUAC will be collected today and in 4, 30, 60 and 180 days from today and for some infants at 365 days from today.

You and your baby’s hemoglobin—A few drops of blood will be collected from you and your baby four days from today to measure the level of hemoglobin, a molecule that helps transport oxygen in your blood. These drops of blood (0.2 mL) will be collected by heelstick for your baby and by finger prick for you.

Your baby’s skinfold thickness—The thickness of your baby’s upper arm will be measured at 180 days (6 months) and for some infants at 365 days (1 year).

Survey—The study nurse will ask you today and in 4, 30, 60 and 180 days about your baby’s health and dietary intake including breastfeeding and other diet, and about your health and your household.

Breast milk volume—Breast milk volume will be assessed for some study mothers and babies using the deuterium oxide method. For this method, you will receive a small volume of deuterium oxide to consume diluted in a beverage 60 days after birth. If you are one of the mothers who receive deuterium oxide, your urine will be collected three times in the first week after receiving deuterium oxide, and your baby’s urine will be collected daily for seven days following administration and then on days 10, 13 and 16 following administration. If use of the deuterium oxide method is not possible due to pandemic- related conditions, breast milk volume will be assessed for some study mothers and babies using the “test weight” method, which will involve weighing your baby before and after breastfeeding.

Stool—your baby’s stool will be collected now, and when the baby is 30 days (approximately 1 month) and in 180 days (approximately 6 months) old. Disposable diapers will be supplied by the study for stool collection.

Breast milk—When the baby is 30 days old, you will be asked if you are willing to hand express 20 mL milk. You will be asked to sign a separate specimen consent form to allow this milk to be stored at the University of California, San Francisco.

Brain imaging—Brain images (pictures) will be obtained for about half (50%) of the infants using a portable magnetic resonance imaging (MRI) device (Hyperfine Swoop MRI) at 3, 6 and 12 months and a fixed MRI device at 12 months. Infants will be selected for brain images based on convenience. The fixed MRI will be done once at Nsambya Hospital when the infant is 12 months old, while the portable MRI will be done at 3, 6 and 12 months at our research clinic. During the MRI, the infant lies quietly on a table that gently slides the infant into a machine that can obtain the images. For the portable MRI, you may have to hold the infant still for 10-12 minutes. The total additional time commitment will be 1 hour per examination, or up to 2 hours at the 12-month visit for both examinations. You will be asked whether you are willing to participate in brain imaging, and participation in brain imaging can be stopped at any time. If there are any abnormal findings, they will be communicated to you within 2-4 weeks by Dr. Nankabirwa or Dr. Murungi who will notify your infant’s regular provider of care or refer you for care at Mulago National Hospital.

Bayley Scales of Infant and Toddler Development—Trained staff will administer the Bayley Scales at 6 and 12 months to learn how your infant’s brain functions.

The calendar of study procedures will be:

**Day 0 (today):** You will be asked to give informed consent for you and your baby. If you give informed consent, study staff will obtain your contact information, collect survey information, measure and weigh you and your baby and collect your baby’s stool if available. If your baby weighs <2500g, you will be assigned either to breastfeed with formula supplementation for 30 days or to breastfeed exclusively with no additional foods or fluids for 6 months.

**Day 4:** Your baby will be weighed and measured. For mothers and babies not yet assigned to either breastfeeding with formula or to exclusive breastfeeding, the baby’s weight will determine whether you and your baby exit the study or become assigned either to breastfeed with formula

supplementation until your baby is 30 days old or to breastfeed exclusively with no additional foods or fluids for 6 months. If you and your bay do not exit the study, the study team will collect survey information including dietary survey and adverse events and will collect hemoglobin from you and your baby.

**Day 14:** The study team will weigh and measure your baby and collect survey information including dietary survey and adverse events.

**Day 30:** The study team will weigh and measure your baby and will collect a stool specimen from your baby, a milk specimen from you and survey information including dietary survey and adverse events.

**Day 60:** The study team will weigh and measure your baby, collect a stool specimen from your baby and collect survey information including dietary survey and adverse events.

**Day 90 (3 months):** The study team will weigh and measure your infant and collect survey information including dietary survey and adverse events. For some infants, the study team will measure brain function with the Bayley and measure brain volume with Hyperfine portable MRI.

**Day 180 (6 months):** Day 30: The study team will weigh and measure your baby, collect a stool specimen from your baby and collect survey information including dietary survey and adverse events. You will then exit the study. For some infants, the study team will measure brain function with the Bayley and measure brain volume with Hyperfine portable MRI.

**Day 365 (12 months):** The study team will weigh and measure your infant and collect survey information including dietary survey and adverse events. For some infants, the study team will measure brain volume with Hyperfine portable MRI and with fixed MRI and measure brain function with the Bayley. You will then exit the study.

During these procedures, it is important to 1) ask questions as you think of them, 2) follow the instructions of the PRIMES study staff and 3) tell the PRIMES study staff if you change your mind about participating in the research study. While participating in this study, you and your baby should not take part in any other research study.

# Who will participate in the study:

This study will enroll mothers and their newborn babies. Mother-newborn pairs will be eligible if the baby weighs 2000-2885 g, and if the mother is years old, intends to breastfeed, has a negative HIV test, lives in the area and anticipates availability for all study visits. Mother- newborn pairs are not eligible (excluded) if the baby is a twin or other multiple, has major congenital anomalies or World Health Organization danger signs, or is enrolled in another study, or if the mother is unable or unwilling to complete all aspects of the protocol or has had another baby enrolled in PRIMES. We expect 324 mother-newborn pairs to participate in this study.


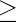


# Possible risks, discomforts, and inconveniences:

For babies randomly assigned to the INTERVENTION, there may be an increased risk of diarrhea, infectious disease and breastfeeding problems, which might increase the baby’s risk of adverse events, hospitalization or death. For babies randomly assigned to the CONTROL, there may be an increased risk of growth failure, which might increase the baby’s risk of adverse events, hospitalization or death. All babies who become ill during participation in PRIMES will be referred by the study physicians to the best available care.

A few drops of blood will be collected by heelstick for babies and finger prick for mothers. This may cause pain or infection. However, great care will be taken to minimize any chance of infection. Participating in this study can take time out of your regular workday, and answering some of these questions may make you uncomfortable. You may also feel uncomfortable during hand expression of breast milk.

Claustrophobia is feeling like you are in a tight or crowded space and is a risk of magnetic resonance imaging (MRI). Great care will be taken to minimize the risk of claustrophobia for infants, including conducting the examination while the infant is sleeping. During Hyperfine portable MRI examinations, you will be encouraged to hold your infant during the examination. During fixed MRI examinations, study staff will stop the examination if your infant appears distressed. Other risks of fixed MRI include hearing risk and risk of metal objects being attracted to the magnet and hitting you or the baby. To reduce hearing risk, your infant will be provided with ear protection during fixed MRI. To reduce the risk of items being attracted to the magnet and hitting you or the baby, fixed MRI will be conducted at Nsambya Hospital by a trained radiologist experienced with techniques for confirming that no metal objects are present.

# Potential benefits:

Your baby may benefit from close checking of growth during the first 180 days. There may also be a public health benefit to understanding infant health in Uganda.

# Confidentiality:

Personal information will be collected about you and your baby, and it will be kept strictly in confidence. Only the authorized study personnel will have access to you and your baby’s medical records and personal identifiers. You will not be identified when the findings of this study are published. All the information you share will be kept in a locked filing cabinet or on secure computer password protected servers that only PRIMES investigators and study staff will have access to.

We will take all necessary measures to make sure that your personal information is kept private. However, we cannot promise total privacy. Your personal information may be given out if required by law. Organizations and people who check the study such as UCSF, the Research and Ethics Committee of Makerere University, and Makerere University research collaborators may see the records that identify you. This may include the informed consent form that you signed.

The records in this study may also be reviewed to make sure that all rules have been followed. If you withdraw at any time for any reason, all measurements and survey responses already collected will be used in the study and kept for up to 10 years. After 10 years, the information will be destroyed unless an extension is approved by the UCSF Institutional Review Board and your institutions Ethical Review Committee, Institutional Review Board or Institutional Review Committee.

# Alternatives:

Joining this study is voluntary; you do not have to join the study if you do not want to. Refusal to join the study will not place you or your baby at risk of losing access to medical care. If you choose to not join the study or if you decide to leave the study, you will continue to receive the exact treatment offered by the responsible authorities.

# Cost:

There will be no cost to you and/or your baby for the study process. The study will pay for those services, supplies, procedures, and care associated with this study that are not a part of your routine healthcare.

# Compensation for participation in the study/ Reimbursement:

You will receive 20,000 Ugandan shillings as reimbursement to cover your transport costs each time you come from home to the study clinic with your baby for investigation. You will also receive three disposable diapers each time urine or stool collection is scheduled for your baby, to allow easy collection of urine and stool.

There is no compensation or monetary payment for participating in this study.

# Questions about the study:

If you ever have questions about this study, need any more information, or if you have a health problem or are hurt in the study, you should contact study Principal Investigator, Dr. Victoria Nankabirwa at telephone number 0755757460.

If you have any questions, you have a right to ask Dr. Nankabirwa or PRIMES study staff. Your questions should be answered clearly and to your satisfaction. You should not feel that you have to participate. If you decide not to participate, tell PRIMES study staff.

Researchers may learn new things in the study that will influence your decision to continue to participate. The researchers will let you know if they learn anything that might make you change your mind about you and your baby continuing to participate in the study.

# Questions about you and your baby’s rights and contact information:

If you have questions about your rights as a research volunteer, you may contact the School of Public Health Higher Degrees Research and Ethics Committee (HDREC) chair Dr. Suzanne Kiwanuka on telephone number 0772886377. The study staff will be happy to help you contact the right person to answer any questions you have.

If you have any questions, you may ask them now or later, even after the study has started. If you wish to ask questions later, you may contact your local Principal Investigator Dr.Victoria Nankabirwa at telephone number 0755757460 or Dr. Joan Murungi at telephone number 0701951550 .

# Statement of voluntariness:

Your and your baby’s participation in this study is entirely voluntary. If you decide to have you and your baby participate, you will be asked to sign this consent form and you will receive a copy with important information such as names and phone numbers of the study team.

You do not have to participate in this research study. It is your choice. You may agree to participate now and change your mind later and stop at any time for any reason. If you choose to stop, this will not affect your relationship with any of the researchers, your healthcare providers, or the Ministry of Health. If you do not wish to participate, you will continue to receive regular healthcare for you and your baby in the same way you did before. If you wish to participate in this study, you must sign this form.

# Withdrawal from study:

You may ask to be removed from the study for any reason at any time. The PRIMES study staff may also remove you or your baby from the study at any time without your consent for any of the following reasons: It is important for your or your baby’s safety; It is not in your or your baby’s best interest; You did not give an accurate history; or You failed to follow the rules of the study or the instructions of PRIMES study staff. If you want to be removed or want your baby to be removed from the study, or if you or your baby are removed from the study, any information collected from you before you were removed from the study will still be used.

# Dissemination of results:

Study findings will be presented to local stakeholders and published in medical journals.

# Ethical approval:

This study has been approved by Makerere University School of Medicine Research and Ethics Committee, the Uganda National Council for Science and Technology and the University of California, San Francisco Institutional Review Board.

# Consent:

Your signature or thumbprint below means that you understand the information given to you about the study and in this consent form. If you sign or place your thumbprint on this form, it means that you agree that you and your baby can be screened to take part in the study. You are not giving up any of your and your child’s legal rights by signing this informed consent document.

**STATEMENT OF CONSENT/ASSENT**

........................................................................... has described to me what is going to be done, the risks, the benefits involved and my rights regarding this study. I understand that my decision to participate in this study will not alter my usual medical care. In the use of this information, my identity will be concealed. I am aware that I may withdraw at anytime. I understand that by signing this form, I do not waive any of my legal rights but merely indicate that I have been informed about the research study in which I am voluntarily agreeing to participate. A copy of this form will be provided to me.

Name ………………………Signature/thumb print of participant Date ……

Name ………………………Signature of parent/guardian for minors (If applicable)……Date

……...

# Declaration by trial team member taking informed consent

I explained the information in this document to the child’s mother.

I encouraged her to ask questions and took adequate time to answer them.

I am satisfied that she adequately understands all aspects of the research, as discussed above.

Name Signature of interviewer/Person obtaining informed consent

……………………Date ………………….

# Declaration of Impartial witness

**Only required if child’s mother or legally appointed representative is illiterate or if required by another party**

I have read the entire Informed Consent Form.

I was present when the study team member obtaining consent read and explained the information in this document to the child’s mother.

The information provided to the child’s mother is accurate and complete. That the child’s mother was encouraged to ask questions.

I am satisfied that the child’s mother fully understands the content of this informed consent document and has had all her questions satisfactorily answered.

Name………………………Signature of witness (if applicable)……………Date…………….

1. **Guinea-Bissau screening and Enrolment consent form**

# INFORMED CONSENT FOR RESEARCH PARTICIPATION

**Participation in the study for mothers and infants**

**Title of proposed study: Preventing infant malnutrition with early supplementation (Prevenção da desnutrição infantil com suplementação precoce, PRIMES)**

**Principal Investigator:** Principal Investigator: Valerie Flaherman, University of California San Francisco and Augusto Braima de Sa, Director of International Partnership for Human Development.

**Co-Investigators:** Susan B Roberts, Augusto Braima de Sa, Amy Ginsburg, Mijung Park, Mi- Ok Kim, Raimundo Co, Alieu Sonko, Anne Wanjuhi and Victoria Laleau

**National doctors of the study:** Carlito Bale, Pediatric and Luis Camala, Neonatologist

**Introduction to research studies:** You are invited to participate in an investigative study conducted by the University of California in San Francisco in partnership with the International Partnership for Human Development (IPHD) in Guinea Bissau. IPHD is also supported by the Ministry of Public Health as they work in recruitment hospitals in Bissau. You may refuse to participate in this study or stop at any time for any reason. If you decide to stop, it will not affect your relationship with any of the researchers, the Ministry of Health, your health care provider or IPHD. If you decide to participate, you will be asked to sign this informed consent form and you will also receive a signed copy. This study was reviewed and approved by the Institutional Review Board of the Ministry of Health of Guinea-Bissau.

**Justification / purpose of the study:** The study aims to determine whether using small amounts of formula combined with breastfeeding may affect the growth of infants during the first month of age.

**Procedures:** If you decide to participate, you and your infant will be measured today and at 4 days of age and have your hemoglobin level checked. Depending on your infant’s weight today and at 4 days of age, the study team may, by chance, assign you and your infant to breastfeeding with 59 mL infant formula daily for the first 30 days after birth, or to standard care feeding which is exclusive breastfeeding without additional food or liquids for 6 months. This designation will occur by chance for 324 infants, with a 50% chance of being assigned to breastfeeding combined with formula supplementation, and a 50% chance of being assigned to exclusive breastfeeding. If you and your infante are assigned to breastfeeding combined with formula supplementation or to breastfeeding exclusively, your infant will be evaluated today and at 4, 14, 30, 60 and 180 days of age. The assessment will include weight, length, arm circumference (MUAC), head circumference, skinfold measurement, food research and assessment of adverse outcomes. Today and at 30 and 180 days of age, infant feces will also be collected. At 30 days of age, we will ask if you could provide 20 mL of breast milk. For some mothers, we will also ask you to take a small amount of deuterium oxide and collect urine from you and your infant 10 times during the following 16 days. Participation will end when the infant reaches 180 days of age or earlier, if we identify any concerns about your safety in participating in the study.

**Responsibilities of the participants:** You should ask questions as you think about them, follow the instructions of the PRIMES study team and inform the PRIMES study team if you change your mind about participating in the research study.

**Possible risks, discomforts and inconveniences:** For infants randomly assigned to the INTERVENTION, there may be an increased risk of diarrhea, infectious diseases and breastfeeding problems, which can increase the infant's risk of adverse events, hospitalization or death. For infants randomly assigned to CONTROL, there may be an increased risk of growth failure, which may increase the infant's risk of adverse events, hospitalization or death. All infants who fall ill while participating in PRIMES will be referred by the study doctors to the best care available. A few drops of blood will be collected by heel puncture for infants and finger puncture for mothers. This may cause pain or infection. However, great care will be taken to minimize the likelihood of infection. We will inform you if new information becomes available that may be relevant to your willingness to participate in this study.

**Potential benefits:** Your infant can benefit from a thorough growth check during the first 180 days. If you or your infant needs medical care, Dr. Bale and Camala will facilitate the referral. It can also be a public health benefit to understand infant health in Guinea-Bissau.

**Alternatives:** Adherence to this study is voluntary; the alternative is to receive your usual care.

**Compensation for participation in the study / Payment:** You will receive three disposable diapers each time urine or feces collection is scheduled for your infant. There is no cash compensation for the participation in this study. Your infant will have the opportunity to be accompanied by the neonatologist and pediatricians for 180 days

**Cost:** There are no costs to participate in this study.

**Privacy and confidentiality:** Researchers will protect your confidentiality as they usually do in research studies. We will make every effort to keep your personal information private and confidential, but this cannot be fully guaranteed. Certain government agencies, such as the Guinea-Bissau Ministry of Health, regulatory authorities and monitors, may request access to the records that identify you. This may include the informed consent form you signed. The records of this study may also be reviewed to ensure that all rules and guidelines have been followed.

You will not be identified when the results of this study are published.

**Early termination:** You or your infant can be withdrawn from the study by the PRIMES study team at any time if it is important to you or your infant's safety, if it is not of interest to you, if you have not provided an accurate history or if you do not follow the rules of the study or the instructions of the PRIMES study team.

# Consent:

Your signature or fingerprint below means that you understand the information provided to you about the study and this consent form. If you sign or place your fingerprint on this form, it means that you agree that you and your infant can be examined to participate in the study. You are not giving up any of your and your infant's legal rights by signing this informed consent document. If you have any concerns or questions, or for more information about the trial or your rights, contact Augusto Braima da Sá (Tel: 245 95 524 08 08) or Raimundo Co (245 95 531 32 61/96

661 24 75).

**Consent documentation**

I received a copy of this form. I read the form, or the form was read to me. I understood the information and had my questions answered satisfactorily. I agree to participate in this study, including giving permission for my infant to participate.

I understand that I will be informed of any new findings developed during this study that may affect my willingness to remain in this study.

Date Signature or fingerprint of mother or father

I fully explained the nature and purpose of the study described above, and the risks involved in its performance. I answered all the questions in the best possible way.

Date and Signature of the Principal Investigator or Representative Signature of the witness

Date and signature of the witness (ACS or midwife)

1. **Infant dietary survey**

| MATERNAL INFANT FEEDING QUESTIONS  ***The following questions pertain to the infant enrolled in the study. Please make sure the mother’s responses are indicated for the enrolled infant.***  Eat & Drink **Between yesterday when you woke up and today when you woke up, has your baby breastfed or received breast milk?**  YES  NO **Between yesterday when you woke up and today when you woke up, how many times did you baby breastfeed?**    Number of times breastfeed **Between yesterday when you woke up and today when you woke up, what did your baby EAT OR DRINK?*****Please ask as an open-ended question; do not read the list. Check all that apply*** Breast milk  Infant formula  Milk  Cow Milk  Goat Milk  Other Milk. Please specify________________  Plain water  Sweetened or flavored water  Fruit juice  Tea or infusion  Oral rehydration salt (ORS) solution  Other liquids  Please specify________________  Other solid or semi-solid food  Please specify________________  ***Proceed to the next page***  Taste **Between yesterday when you woke up and today when you woke up, what did your baby TASTE?*****Please ask as an open-ended question; do not read the list. Check all that apply*** Breast milk  Infant formula  Milk  Cow Milk  Goat Milk  Other Milk. Please specify________________  Plain water  Sweetened or flavored water  Fruit juice  Tea or infusion  Oral rehydration salt (ORS) solution  Other liquids  Please specify________________  Other solid or semi-solid food  Please specify________________  Eat & Drink-last study visit **Since the last study visit, what did your baby EAT OR DRINK?*****Please ask as an open-ended question; do not read the list. Check all that apply*** Breast milk  Infant formula  Milk  Cow Milk  Goat Milk  Other Milk. Please specify________________  Plain water  Sweetened or flavored water  Fruit juice  Tea or infusion  Oral rehydration salt (ORS) solution  Other liquids  Please specify________________  Other solid or semi-solid food  Please specify________________  ***Proceed to the next page***  Taste-last study visit **Since the last study visit, what did your baby TASTE?*****Please ask as an open-ended question; do not read the list. Check all that apply*** Breast milk  Infant formula  Milk  Cow Milk  Goat Milk  Other Milk. Please specify________________  Plain water  Sweetened or flavored water  Fruit juice  Tea or infusion  Oral rehydration salt (ORS) solution  Other liquids  Please specify________________  Other solid or semi-solid food  Please specify________________  Vitamins & Minerals **Since the last study visit, what vitamins and minerals has the infant had?** ***Please ask as an open-ended question; do not read the list. Check all that apply*** Vitamin A  Iron & Folic Acid  Calcium & Phosphorus  Vitamin D  Zinc  None  Other  Please specify________________  ***Proceed to the next page***  Infant Health **Has your infant had blood in stool since the last study visit?**  YES  NO **Has your infant had profuse/watery diarrhea since the last study visit?**  YES  NO **Has your infant had fever since the last study visit?**  YES  NO **Has your infant had low body temperature since the last study visit?**  YES  NO **Has your infant had vomiting since the last study visit?**  *Vomiting is generally forceful, and plentiful, babies usually show signs of distress when vomiting, which can be associated with crying and painful squirming. Babies rarely react to spitting up.*  YES  NO **Has your infant had cough since the last study visit?**  YES  NO **Has your infant had difficulty breathing since the last study visit?**  YES  NO **Has your infant had jaundice since the last study visit?**  ***Proceed to the next page***  YES  NO **Has your infant had convulsions or seizures since the last study visit?**  YES  NO **Has your infant had lethargy or decreased consciousness since the last study visit?**  YES  NO **Does your infant have Sickle Cell disease?**  YES  NO  Unknown **Does your infant have tuberculosis?**  YES  NO  Unknown **Has your infant had any other symptoms since last study visit?**  YES  NO **Has your infant received any medication since the last study visit?**  YES  Antibiotic. Please specify ______________  Other. Please specify ______________  NO  Unknown **Is your infant currently in an outpatient feeding program?**  YES  NO **Has your infant been hospitalized since last study visit?** YES. Please specify ______________  NO  ***Proceed to the next page***   **Was data collection from Day 30 completed?** Completed all data collection  Partial data collection only  Did not complete data collection  Other  Please specify ______________  Notes |  |
| --- | --- |
